# Ccdc103 promotes myeloid cell proliferation and migration independent of motile cilia

**DOI:** 10.1242/dmm.048439

**Published:** 2021-05-24

**Authors:** Lauren G. Falkenberg, Sarah A. Beckman, Padmapriyadarshini Ravisankar, Tracy E. Dohn, Joshua S. Waxman

**Affiliations:** 1 Medical Scientist Training Program, University of Cincinnati College of Medicine, Cincinnati, OH 45267, USA; 2 Molecular and Developmental Biology Graduate Program, University of Cincinnati College of Medicine and Cincinnati Children's Hospital Medical Center, Cincinnati OH 45267, USA; 3 Molecular Cardiovascular Biology Division and Heart Institute, Cincinnati Children's Hospital Medical Center, Cincinnati, OH 45229, USA; 4 Department of Pediatrics, University of Cincinnati College of Medicine, Cincinnati, OH 45267, USA

**Keywords:** CCDC103, Cell migration, Microtubules, Myeloid cells, Primary ciliary dyskinesia, Zebrafish

## Abstract

The pathology of primary ciliary dyskinesia (PCD) is predominantly attributed to impairment of motile cilia. However, PCD patients also have perplexing functional defects in myeloid cells, which lack motile cilia. Here, we show that coiled-coil domain-containing protein 103 (*CCDC103*), one of the genes that, when mutated, is known to cause PCD, is required for the proliferation and directed migration of myeloid cells. CCDC103 is expressed in human myeloid cells, where it colocalizes with cytoplasmic microtubules. Zebrafish *ccdc103*/*schmalhans* (*smh*) mutants have macrophages and neutrophils with reduced proliferation, abnormally rounded cell morphology and an inability to migrate efficiently to the site of sterile wounds, all of which are consistent with a loss of cytoplasmic microtubule stability. Furthermore, we demonstrate that direct interactions between CCDC103 and sperm associated antigen 6 (SPAG6), which also promotes microtubule stability, are abrogated by *CCDC103* mutations from PCD patients, and that *spag6* zebrafish mutants recapitulate the myeloid defects observed in *smh* mutants. In summary, we have illuminated a mechanism, independent of motile cilia, to explain functional defects in myeloid cells from PCD patients.

This article has an associated First Person interview with the first author of the paper.

## Abstract (precis)

**Editor's choice:** We show that Ccdc103 regulates myeloid migration and proliferation independent of motile cilia in zebrafish embryos, and that CCDC103 mutations underlying primary ciliary dyskinesia abrogate interactions with the microtubule-stabilizing protein SPAG6.

## INTRODUCTION

Primary ciliary dyskinesia (PCD), a disorder defined by impairment of motile cilia, occurs in 1:10,000-40,000 live births (Damseh et al., 2017). A genetically and phenotypically heterogeneous disease, PCD often presents with recurrent respiratory infections due to impaired mucociliary clearance, as well as organ laterality randomization and infertility (Leigh et al., 2009; Horani et al., 2016; Shapiro et al., 2018). Although most of the phenotypic hallmarks of PCD are attributable to organs that require motile cilia, it has also been hypothesized that an additional primary immune dysfunction might contribute to the chronic lung infections seen in PCD patients. Intriguingly, although myeloid cells have never been shown to possess motile cilia (Finetti et al., 2009), studies spanning multiple decades, most of which antedate any identification of genetic lesions in PCD patients, report that myeloid cells isolated from PCD patients have functional defects, including disrupted migration in response to chemotactic stimuli and dysregulated expression of surface receptors (Afzelius et al., 1980; Cockx et al., 2018; Englander and Malech, 1981; Valerius et al., 1983; Walter et al., 1990). Additionally, ultrastructural analysis coincident with these neutrophil migration studies showed disruption of cytoplasmic microtubules, including altered number and location of centrioles (Valerius et al., 1983). These observations, although made in multiple independent cohorts of patients with variable and unknown genotypes, have never been mechanistically explained. Thus, understanding the molecular basis for defects in PCD patient myeloid cells may provide critical insights into this disease process and open new avenues for potential therapies to improve outcomes for what is a lifelong and often debilitating condition.

Presently, mutations in more than 40 different gene products have been implicated in PCD (Horani et al., 2016). The genes most commonly mutated in this disorder include those coding for light-, intermediate- and heavy-chain subunits of axonemal dynein and their associated chaperones/assembly factors required for ciliary motility, including dynein axonemal assembly factor 1 (DNAAF1), and microtubule-binding proteins such as sperm-associated antigen 1 (SPAG1) and SPAG17 (Knowles et al., 2013; Tarkar et al., 2013; Horani et al., 2016; Abdelhamed et al., 2020). One of these axonemal dynein assembly factors is coiled-coil domain-containing protein 103 (CCDC103). Mutations in *CCDC103* underlie ∼4% of all PCD cases, but in certain geographic subpopulations in which PCD is more prevalent it has been shown to be responsible for ∼20% of cases (Shoemark et al., 2018). Human CCDC103 is a small 242-amino acid (AA) protein that consists of conserved central RPAP3_C and flanking N- and C-terminal coiled-coil domains (King and Patel-King, 2020). CCDC103 is critical for the proper docking and assembly of the outer dynein arms, which facilitate ciliary motion, but is also found localized throughout the cytoplasm of both ciliated and non-ciliated cells (Panizzi et al., 2012). *In vitro* studies have shown that CCDC103 forms self-organizing oligomers, and that it binds periodically to cytoplasmic microtubules and can facilitate the stability of assembled microtubules (King and Patel-King, 2015, 2020). Despite these studies, it is not yet known whether CCDC103 has cilia-independent requirements in the cytoplasm *in vivo.*

Here, we show that *Ccdc103* has conserved expression in vertebrate myeloid lineages, including primitive macrophages and neutrophils, and localizes with cytoplasmic dynein (DYNH1C1) on microtubules within their cytoplasm. Using zebrafish *ccdc103*/*schmalhans* (*smh*) mutants (Panizzi et al., 2012), an established model for PCD, we find that myeloid cells lacking *Ccdc103* have decreased proliferation, disrupted directed migration to sterile wound sites, and an abnormal spherical morphology, findings which are consistent with a loss of cytoplasmic microtubule stability. Interestingly, we identified sperm-associated antigen 6 (SPAG6), which promotes microtubule stability and is associated with proper proliferation and migration in multiple cells types, as a novel CCDC103-binding partner. Patient mutations in CCDC103 abrogate interactions with SPAG6, while engineered zebrafish *spag6* mutants recapitulate functional defects in myeloid cells found in *smh* mutants. Our study is the first to identify roles for CCDC103 within the cytoplasm, independent of motile cilia, and to illuminate a mechanism underlying unexplained functional defects in PCD patient myeloid cells, which may open new avenues to improve outcomes for these patients.

## RESULTS

### *ccdc103* is expressed in zebrafish myeloid progenitor cells

**Fig. 1.**
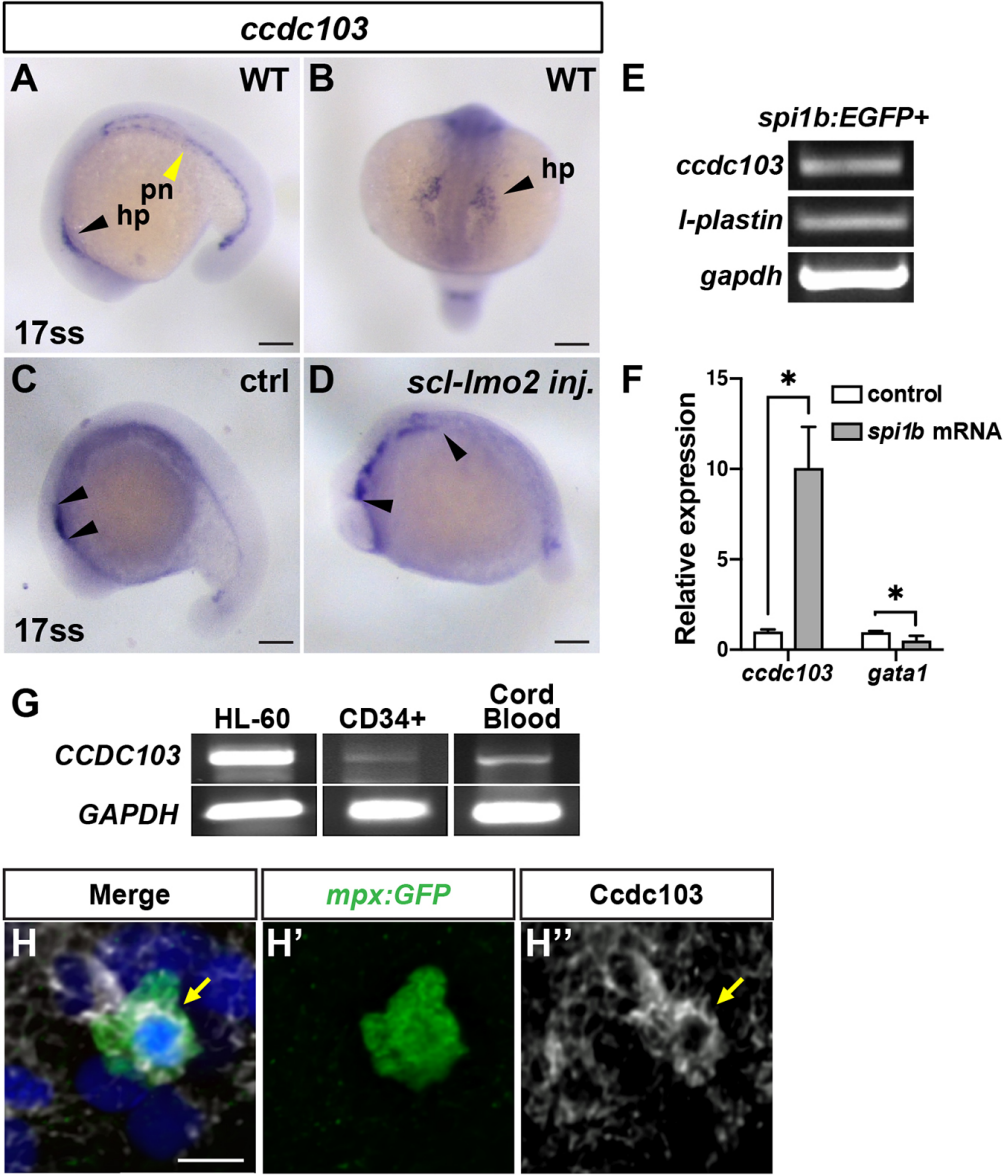
***ccdc103* is expressed in myeloid cells.** (A,B) *ccdc103* expression in a 17-somite stage (ss) wild-type (WT) embryo. hp, hematovascular progenitors (black arrowheads); pn, pronephros (yellow arrowhead). (C,D) The anterior domain of *ccdc103* expression (black arrowheads) is expanded in embryos co-injected with *scl* and *lmo2* mRNAs. In A, C and D, images are lateral views with anterior left. In B, image is a dorsal view. (E) RT-PCR for *ccdc103*, *l-plastin* (positive control) and *gapdh* performed on cDNA isolated from fluorescence-activated cell sorting (FACS)-sorted *spi1b:EGFP*^+^ zebrafish myeloid progenitor cells. (F) RT-qPCR for *ccdc103* and *gata1* performed on cDNA isolated from embryos injected with *spi1b* mRNA. *P<0.05 (two-tailed unpaired Student's *t*-test). (G) RT-PCR for human *CCDC103* was performed with cDNA isolated from HL-60 cells, CD34^+^/CD38^–^ hematopoietic stem cells (HSCs) and cord blood. (H-H″) Immunohistochemistry (IHC) for Ccdc103 (arrows), GFP and DAPI in an *mpx:GFP^+^* zebrafish neutrophil at 24 hpf. Scale bars: 100 μm (A-D), 10 μm (H).

In an *in situ* hybridization (ISH) screen for novel genes expressed in the anterior lateral plate mesoderm (ALPM), we observed *ccdc103* expression lateral to the developing head in 17-somite stage (ss) embryos (Fig. 1A,B). This expression pattern was surprising, given that in zebrafish this region gives rise to an anterior population of primitive myeloid progenitors and, at the time of this initial observation, Ccdc103 had only been characterized in the context of motile cilia (Panizzi et al., 2012; Austin-Tse et al., 2013; Casey et al., 2015). As previously reported (Panizzi et al., 2012), *ccdc103* was also highly expressed in the pronephros (Fig. 1A), an organ that requires motile cilia.

As there is some heterogeneity in the progenitor cell types in the ALPM (Gering et al., 2003), we initially determined whether *ccdc103* is expressed in myeloid progenitors by co-injecting mRNA encoding the master hematopoietic regulators *scl* (also known as *tal1*) and *lmo2*. We found that the anterior *ccdc103* expression domain was expanded in injected embryos (Fig. 1C,D). Reverse transcription PCR (RT-PCR) for *ccdc103* in flow-sorted *spi1b:EGFP^+^* cells from 17-ss embryos further supported its expression in myeloid progenitors (Fig. 1E). Additionally, real-time quantitative PCR (RT-qPCR) performed on complementary DNA (cDNA) from whole zebrafish embryos injected with the pro-myeloid pioneer factor *spi1b* showed a significant increase in *ccdc103* expression compared to controls, along with a corresponding decrease in *gata1* (also known as *gata1a*) expression, denoting a shift away from an erythroid fate (Fig. 1F) (Ward et al., 2003). Furthermore, RT-PCR also detected *CCDC103* expression in human myeloid cells and myeloid progenitors, whole-blood-derived CD34^+^/CD38^−^ cells, whole cord blood and in the promyelocytic leukemia HL-60 cell line (Fig. 1G; Fig. S1). Thus, our data show that *ccdc103* has previously unrecognized conserved expression in zebrafish and human myeloid cells.

**Fig. 2.**
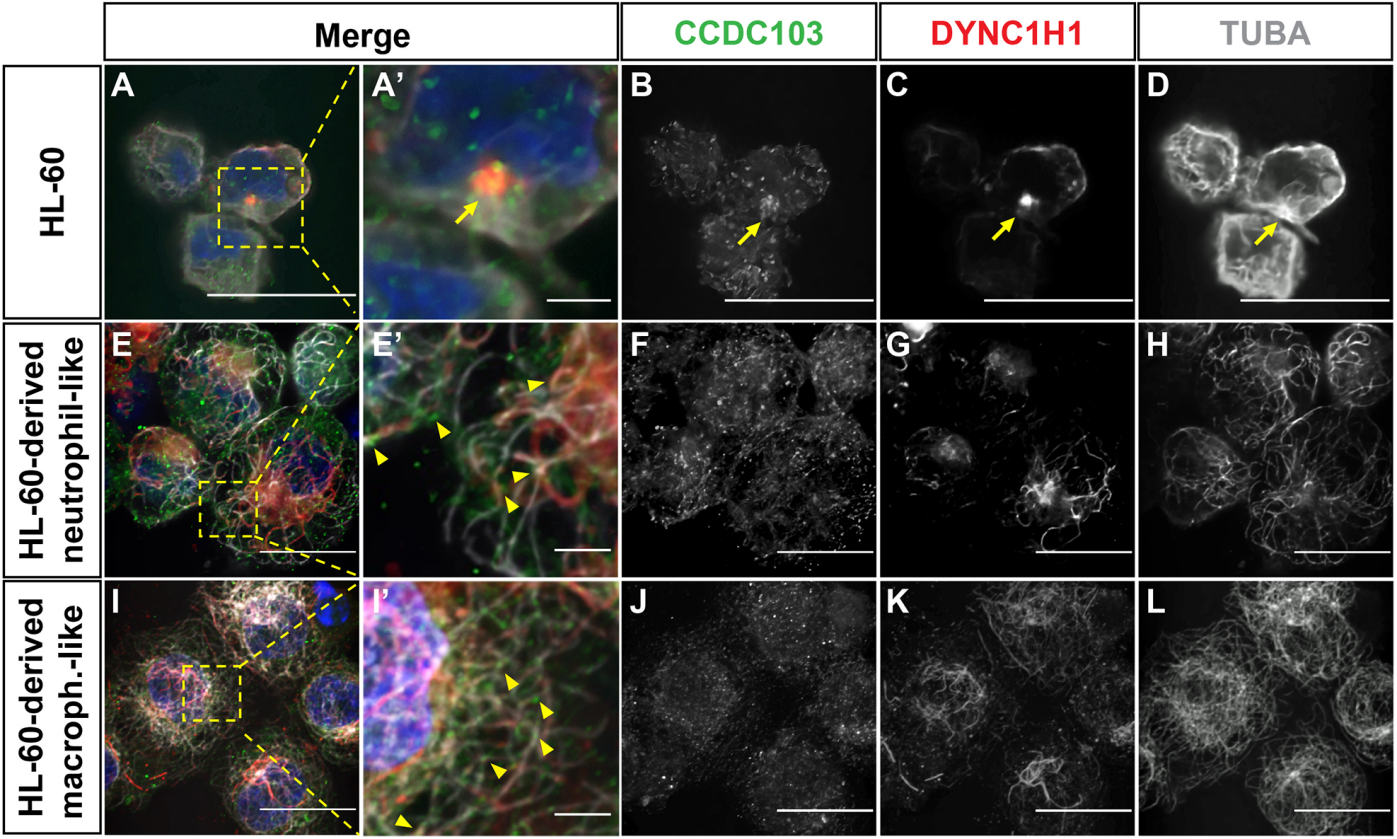
**CCDC103 colocalizes with TUBA and DYNC1H1.** (A-D) IHC for CCDC103, DYNC1H1 and TUBA in HL-60 cells. Yellow arrows indicate the microtubule-organizing center. (E-H) HL-60-derived neutrophil-like cells. (I-L) HL-60-derived macrophage-like cells. In E-L, yellow arrowheads indicate regions of colocalization of CCDC103 and TUBA in neutrophil-like and macrophage-like cells. Scale bars: 10 μm (A-E,F-I,J-L), 2 μm (A′,E′,I′).

Using immunohistochemistry (IHC) with a pan-Ccdc103 antibody, we also confirmed that Ccdc103 is present in primitive zebrafish *spi1b:EGFP^+^* myeloid progenitor cells, where it appeared as puncta that were absent in *smh* embryos (Figs S2 and S3). However, in differentiated *mpx:GFP^+^* primitive neutrophils, it had a perinuclear localization (Fig. 1H-H″). To examine CCDC103 localization in human myeloid cells, we performed immunostaining on undifferentiated HL-60 cells, as well as HL-60-derived macrophage-like and neutrophil-like cells (Breitman et al., 1980; Millius and Weiner, 2010). In the undifferentiated HL-60 myeloid progenitor cells, we observed that CCDC103 localized in larger, more sparse puncta, whereas in differentiated HL-60-derived neutrophils and macrophages, CCDC103 had smaller punctate and a more diffuse distribution throughout the cells (Fig. 2A-B,E-F,I-J). Interestingly, the CCDC103 puncta in all these cells appeared to associate closely with the cytoplasmic microtubule network. Furthermore, in undifferentiated HL-60 myeloid progenitors, CCDC103 and cytoplasmic dynein heavy chain 1 (DYNC1H1) aggregates were concentrated at putative microtubule-organizing centers (MTOCs), as revealed with α-Tubulin (TUBA; also known as TUBA4A in humans) counterstaining (Fig. 2A-D), which was consistent with findings in zebrafish myeloid cells (Fig. 1H,H′). In the differentiated HL-60 cells, CCDC103 was more broadly associated with the microtubule network and DYNC1H1, and the puncta often appeared to sit on top of the microtubules (Fig. 2E-L). Although previous work has indicated that myeloid cells do not have motile or primary cilia (Finetti et al., 2009; Yuan and Sun, 2013; McClure-Begley and Klymkowsky, 2017), a recent study suggested that myeloid cells may have small primary cilia (Singh et al., 2016). Therefore, we performed staining for acetylated (K40) TUBA, which marks cilia, in the undifferentiated HL-60 and differentiated HL-60 derived cells. In both undifferentiated and differentiated HL-60 cells, we saw no indication of primary cilia, although we did observe that acetylated TUBA expression was surprisingly extensive throughout the cytoplasmic microtubule network (Fig. S4). Thus, we find that CCDC103 has conserved expression and colocalizes with cytoplasmic microtubules in vertebrate myeloid cells.

### Myeloid cells in *smh* mutants have reduced proliferation

**Fig. 3.**
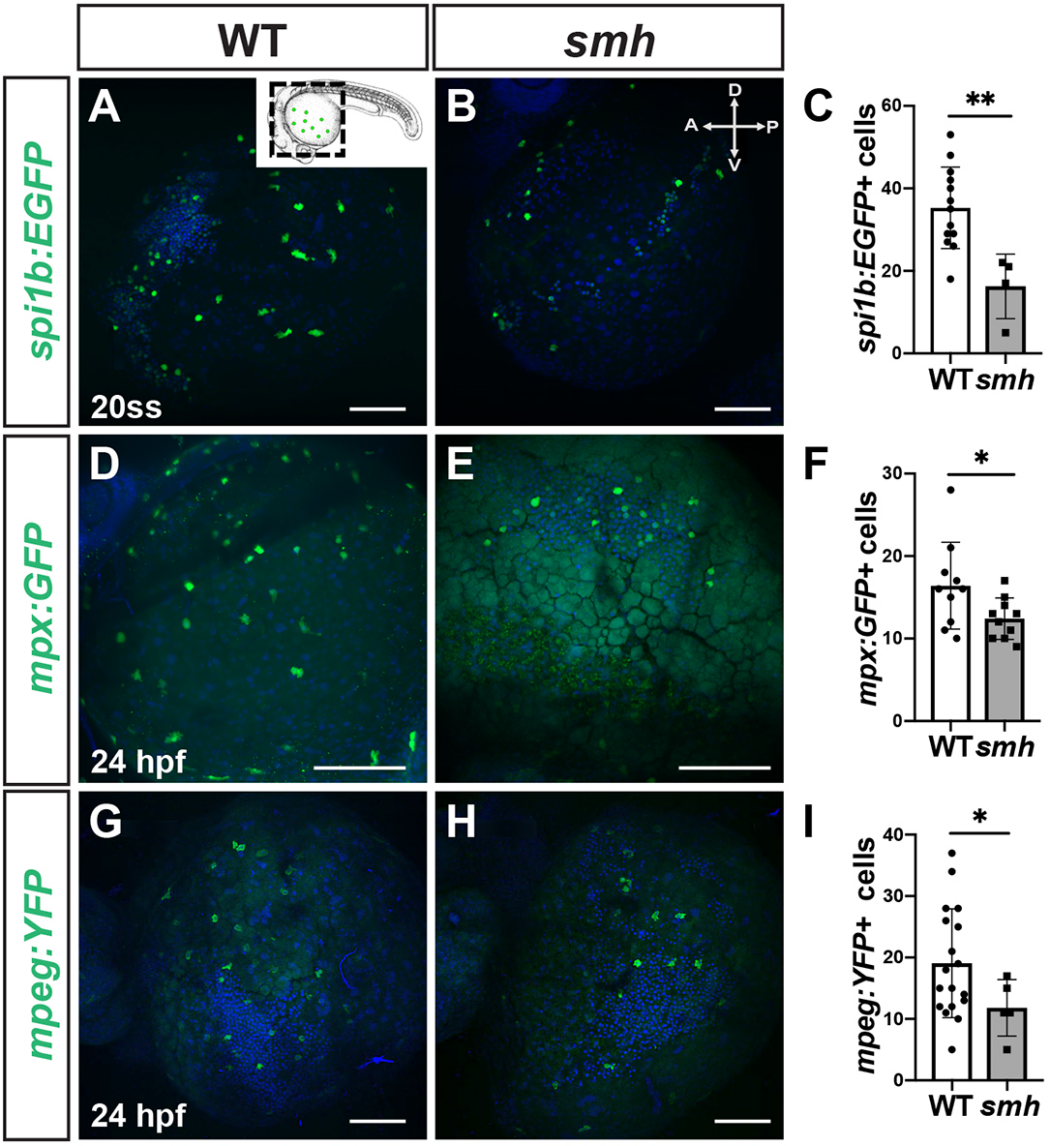
***smh* mutant embryos have fewer primitive myeloid cells at 24 hpf.** (A,B) Representative images of WT sibling (*n*=15) and *smh* embryos (*n*=5) carrying the *spi1b:EGFP* transgene at the 20-ss. Inset indicates the region of the embryo being imaged. A, anterior; D, dorsal; P, posterior; V, ventral. (C) Quantification of *spi1b:EGFP^+^* myeloid progenitors from one yolk hemisphere of individual embryos. (D,E) Representative images of WT (*n*=10) and *smh* (*n*=10) embryos with *mpx:GFP* transgene at 24 hpf. (F) Quantification of *mpx:GFP^+^* cells from one yolk hemisphere of individual embryos. (G,H) Representative images of WT sibling (*n*=19) and *smh* (*n*=9) embryos with *mpeg1.1:YFP* transgene at 24 hpf. (I) Quantification of *mpeg1.1:YFP^+^* myeloid progenitors from one yolk hemisphere of individual embryos. For graphs in C, F and I, each data point represents an individual embryo. **P<0.005, *P<0.05 (two-tailed unpaired Student's *t*-test). Scale bars: 100 μm.

The zebrafish *smh* mutant has ciliary paralysis analogous to that seen in PCD patients and results from a point mutation that causes premature truncation of Ccdc103 (Panizzi et al., 2012). Although requirements for Ccdc103 in cells with motile cilia have been well characterized (Panizzi et al., 2012; King and Patel-King, 2020), previous work had not examined its function in myeloid cells. Given our data showing that Ccdc103 is expressed in primitive myeloid progenitors and differentiated myeloid cells, we first sought to understand whether Ccdc103 is required to promote the development of these cells. Thus, we quantified the number of myeloid progenitors and differentiated macrophages and neutrophils adjacent to the head and on the anterior yolk in wild-type (WT) and *smh* mutant embryos using the *spi1b:EGFP*, *mpeg:YFP* and *mpx:GFP* transgenes. We found that *smh* mutants had fewer of each of these cell types compared to their WT siblings (Fig. 3A-I). These observations were further supported when cells were quantified following whole-mount ISH (Fig. S5). Conversely, embryos injected with *ccdc103* mRNA had an increase in the numbers of *mpx^+^* and *mfap4^+^* cells compared to controls (Fig. S6).

**Fig. 4.**
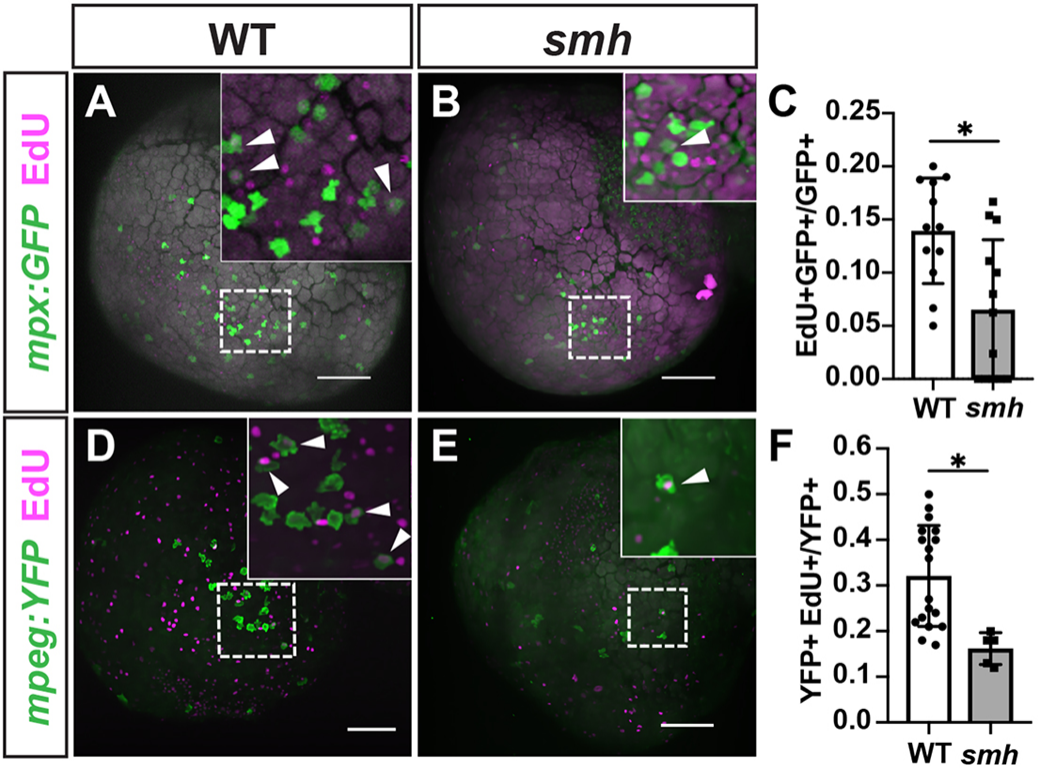
***smh* myeloid cells are less proliferative.** (A,B) Representative images of WT (*n*=12) and *smh* (*n*=19) embryos with *mpx:GFP* transgene pulsed with EdU at the 20-ss and fixed at 24 hpf. White arrowheads indicate EdU^+^ cells. (C) Quantification of EdU^+^/*mpx:GFP^+^* cells from one yolk hemisphere of individual embryos, each data point representing an individual embryo. (D,E) Representative images of WT (*n*=20) and *smh* (*n*=9) embryos with *mpeg:YFP* transgene pulsed with EdU at the 20-ss and fixed at 24 hpf. White arrowheads indicate EdU^+^ cells. (F) Quantification of EdU^+^/*mpeg:YFP^+^* cells from one yolk hemisphere of individual embryos, each data point representing an individual embryo. For C and F, *P<0.05 (two-tailed unpaired Student's *t*-test). Scale bars: 100 μm.

Based on previous biochemical studies showing that CCDC103 is capable of stabilizing microtubules in solution (King and Patel-King, 2015), and because increased microtubule instability is known to affect cell proliferation (Meyvisch et al., 1983), we next determined whether the decrease in these myeloid populations was due to reduced proliferation. *mpx:GFP* and *mpeg:YFP* embryos were pulsed with 5-ethynyl-2′-deoxyuridine (EdU) at the 20-ss, fixed at 24 h post-fertilization (hpf) and imaged for EdU incorporation. We found that *smh* embryos displayed a reduced proportion of EdU^+^/*mpx:GFP*^+^ and EdU^+^/*mpeg:YFP*^+^ cells (Fig. 4A-F), indicating that the decreased numbers of anterior myeloid cells at 24 hpf resulted from decreased proliferation. Collectively, these results indicated that Ccdc103 promotes primitive myeloid proliferation in zebrafish embryos.

### Myeloid cells in *smh* mutants have directed migration and morphology defects

**Fig. 5.**
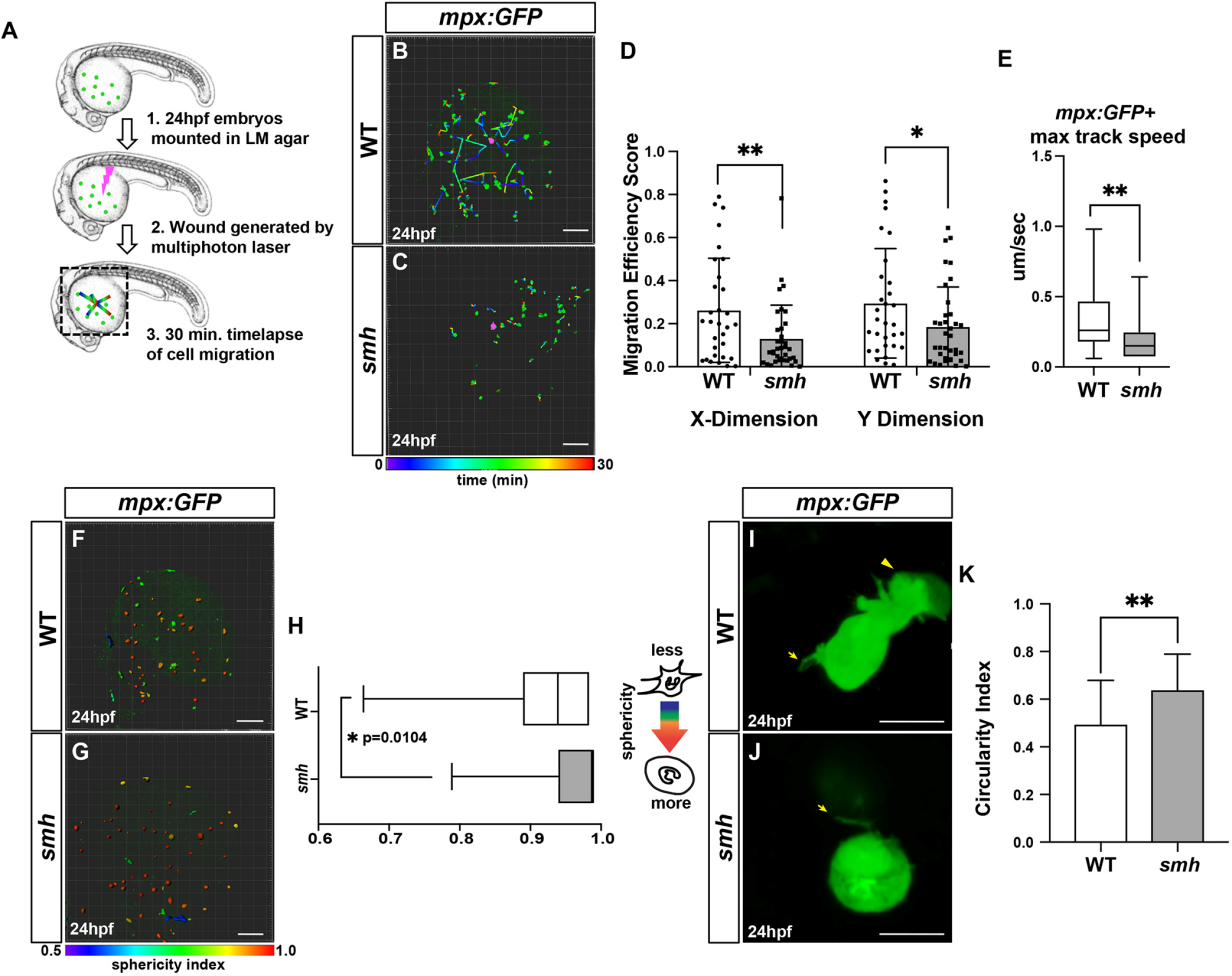
***smh* mutant neutrophils display directed migration defects.** (A) Schematic outlining the wound generated with the multiphoton laser. The dashed line box indicates the imaging area. (B,C) Representative confocal projection images of wounded WT (*n*=6) and *smh* (*n*=6) embryos with the *mpx:GFP* transgene at 24 hpf. (D) Quantification of migration efficiency scores calculated from point position data generated in Imaris. Each data point represents an individual cell from a minimum of three separate experiments, per genotype. (E) Quantification of the maximum track speed of migrating cells. (F,G) Maximum confocal projections of wounded WT and *smh* embryos bearing the *mpx:GFP* transgene with individual cells projected as 3D surfaces and color coded according to sphericity index, as calculated by Imaris. (H) Quantification of the mean cell sphericity index. Box and whisker plots represent individual cells from all independent experiments at each time point imaged, in order to capture changes in cell sphericity over the course of the time-lapse sequence. In E and H, the box and whisker plots represent the median value (center line), with the box including all values from the two median quartiles and the whiskers representing minimum and maximum values (with no outliers excluded). (I,J) Representative single *z*-slices from high-resolution live confocal imaging of individual *mpx:GFP*^+^ cells from WT (*n*=10) and *smh* (*n*=12) embryos. In I, the arrow indicates a uropod and the arrowhead indicates lamellipodia. In J, the arrow indicates cytoplasmic extension. (K) Mean circularity index for individually imaged cells as calculated in Imaris. **P<0.005, *P<0.05 (two-tailed unpaired Student's *t*-test). Scale bars: 100 μm (B,C,F,G), 10 μm (I,J).

Primary neutrophils isolated from PCD patients have impaired directed migration to chemokines in *in vitro* assays (Afzelius et al., 1980; Walter et al., 1990; Kantar et al., 1993; Cockx et al., 2017). Furthermore, microtubule destabilization via nocodazole treatment decreases the ability of neutrophils to migrate appropriately toward a chemical stimulus (Gundersen and Bulinski, 1988; Kadir et al., 2011; Yoo et al., 2012). Thus, we hypothesized that if Ccdc103 is required to promote microtubule stability in myeloid cells, the myeloid cells in *smh* mutants may have impaired migration*.* To determine whether neutrophils from *smh* mutant embryos have defects in directed migration, we generated sterile wounds on the yolks of zebrafish embryos and used time-lapse imaging to track the ability of stimulated neutrophils to home to the site of injury (Fig. 5A), as previously reported (Redd et al., 2006). At 24 hpf, neutrophils from WT sibling *mpx:GFP* embryos homed more effectively to wound sites than neutrophils from *smh* mutants (Fig. 5B,C; Movies 1 and 2), and we calculated significantly decreased migration efficiency in both the X and Y dimensions in *smh* mutants compared to WT controls (Fig. 5D). Furthermore, *smh* neutrophils displayed a significant decrease in their velocity compared to controls, as measured by maximum track speed (Fig. 5E). Similar results were obtained for macrophages in *smh*; *mpeg:YFP* embryos (Movies 3 and 4, Fig. S7). In addition to impaired migration, nocodazole-treated neutrophils display a more spherical morphology than WT neutrophils (Yoo et al., 2010, 2012; Barros-Becker et al., 2017). Reminiscent of these studies, we also observed a higher mean sphericity index in neutrophils from *smh* mutants (Fig. 5F-H). Furthermore, high-resolution time-lapse imaging of individual neutrophils showed that neutrophils from control *mpx:GFP* embryos predominantly adopt a clearly polarized elongated morphology, with defined uropods and lamellipodia characteristic of mature migrating neutrophils (Fig. 5I; Movie 5), whereas neutrophils from *smh*; *mpx:GFP* embryos predominantly lacked this characteristic shape and were rounded with minimal cytoplasmic extensions (Fig. 5J,K; Movie 6).

**Fig. 6.**
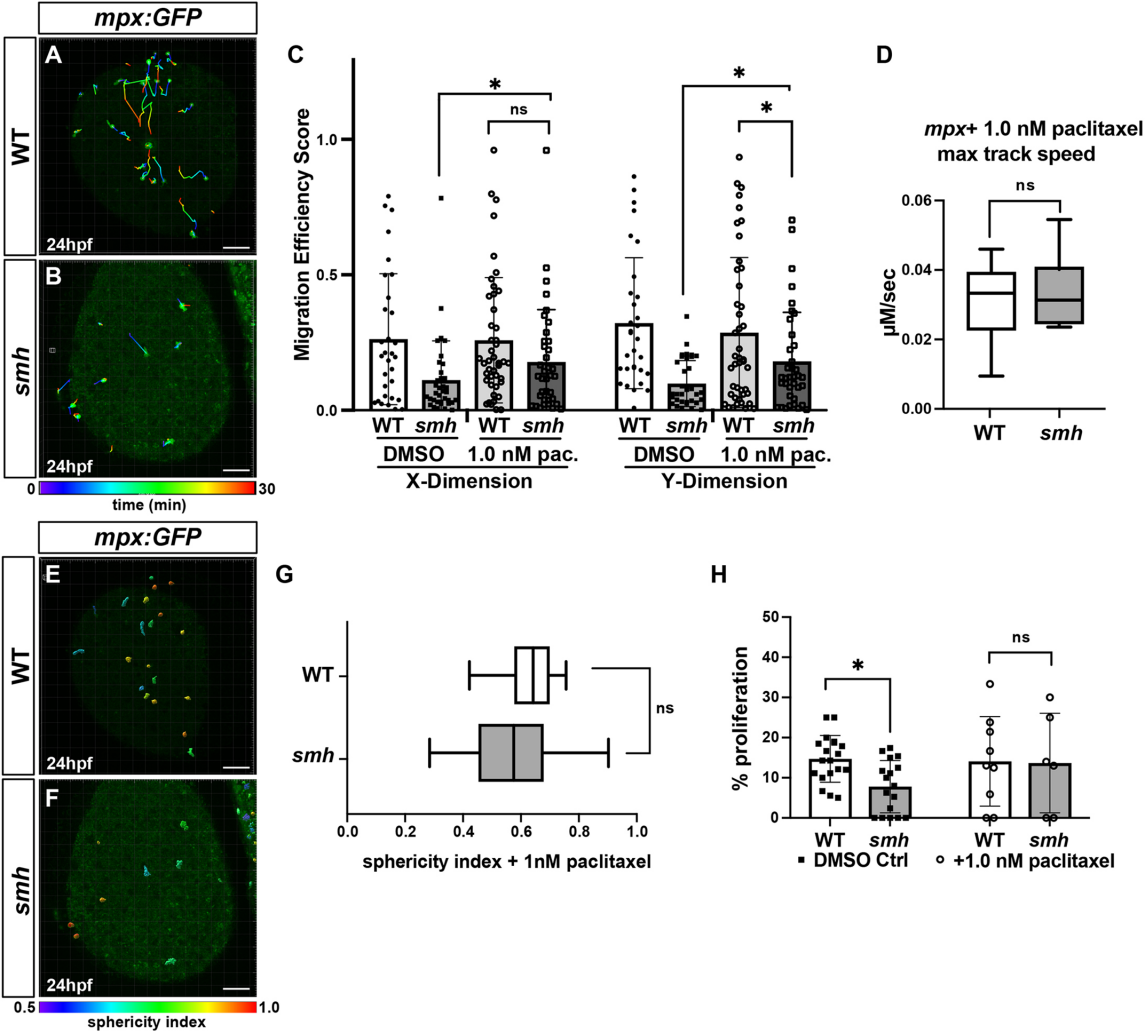
**Paclitaxel can rescue proliferation and migration defects in *smh* mutants.** (A,B) Representative confocal projection images from wounding experiments of paclitaxel-treated WT (*n*=4) and *smh* (*n*=4) *mpx:EGFP* transgenic embryos at 24 hpf. (C) Quantification of migration efficiency scores calculated from point position data generated in Imaris. Each data point represents an individual cell from a minimum of three separate experiments, per genotype, per treatment. (D) Quantification of maximum track speed. Each data point represents an individual cell. Cell tracks and sphericity were generated by Imaris. (E,F) Maximum confocal projections of wounded, paclitaxel-treated WT and *smh* embryos bearing the *mpx:GFP* transgene with individual cells projected as 3D surfaces and color coded according to sphericity index as calculated by Imaris. Red, more spherical; blue, less spherical. (G) Mean cell sphericity indices as calculated in Imaris. In D and G, the box and whisker plots represent the median value (center line), with the box including all values from the two median quartiles and the whiskers representing minimum and maximum values (with no outliers excluded). (H) The percentage of EdU^+^/*mpx:EGFP*^+^ cells from transgenic WT and *smh* mutant embryos, pulsed with EdU at 17-ss and treated with paclitaxel from 17-ss to 24 hpf. *P<0.05; ns, not significant (two-tailed unpaired Student's *t*-test). Scale bars: 100 μm.

Given the correlation of myeloid defects in *smh* mutants to those in embryos with destabilized microtubules, we asked whether the proliferation, directed migration and morphology defects in *smh* myeloid cells could be rescued by paclitaxel-mediated microtubule stabilization. A low dose of paclitaxel that did not cause overt developmental defects was administered to embryos at the 20-ss, followed by sterile wounding assays and EdU pulse-chase experiments. We observed that these low concentrations of paclitaxel were able to provide a partial rescue of the migration defects evident in *smh* embryos (Fig. 6A-D). Furthermore, paclitaxel treatment of *smh* embryos resulted in decreased cell sphericity compared to dimethyl sulfoxide (DMSO)-treated *smh* embryos (Fig. 6E-G; Movies 7 and 8). Additionally, paclitaxel administration rescued the proliferation defects in *smh* mutants (Fig. 6H). Taken together, our data support that Ccdc103-dependent microtubule stability is necessary to promote both myeloid cell proliferation and directed migration.

### CCDC103 interacts with the microtubule-associated protein SPAG6

**Fig. 7.**
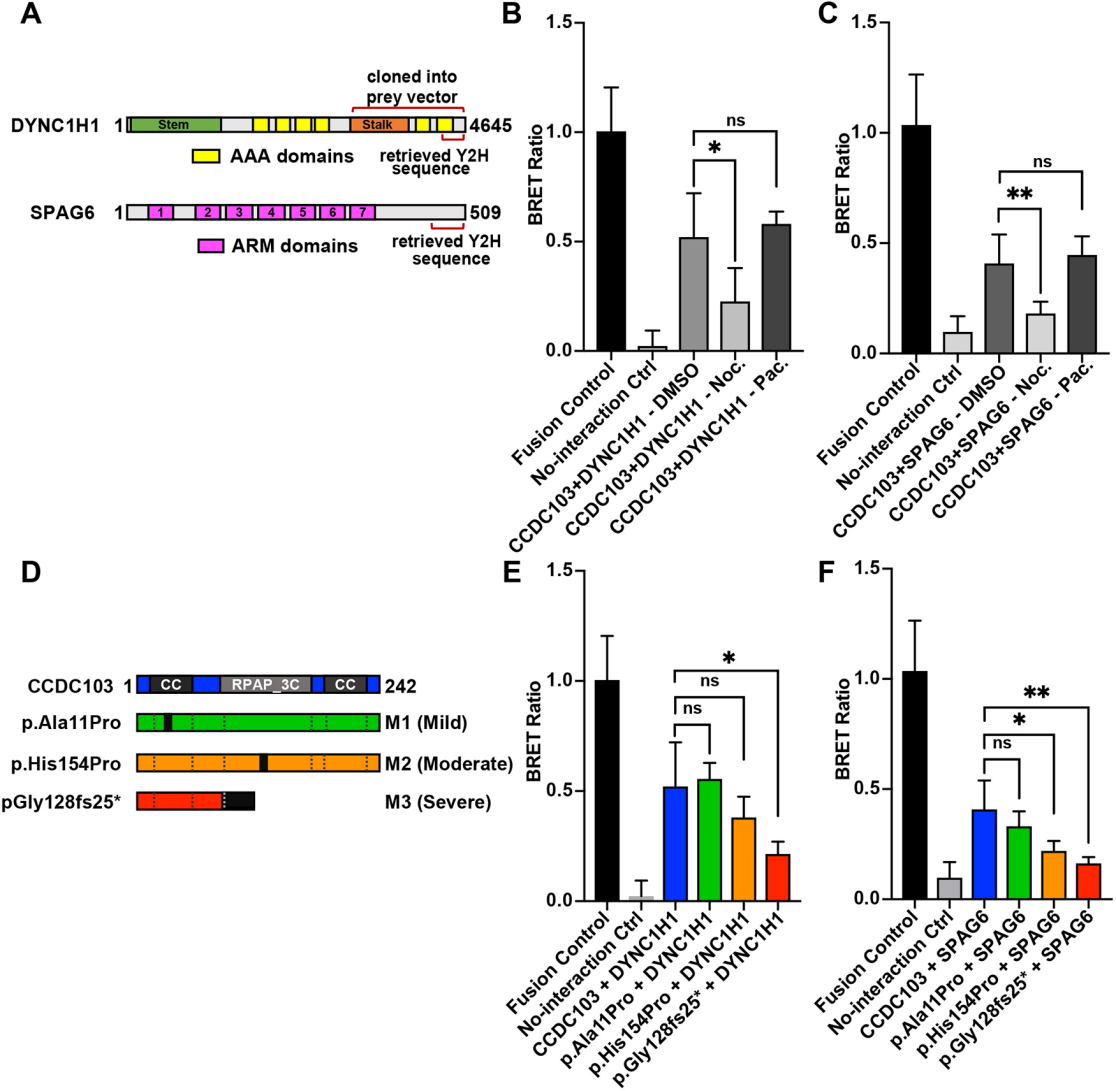
**SPAG6 directly interacts with CCDC103.** (A) Schematic of human SPAG6 and DYNC1H1 proteins. Specific domains identified by Y2H and portion of DYNC1H1 cloned into the LuTHy prey vector are indicated with brackets. The entirety of the *SPAG6* CDS was included in the respective LuTHy prey vector. (B) BRET ratios for the interaction between CCDC103 and DYNC1H1 in the presence of DMSO, nocodazole and paclitaxel. (C) BRET ratios for the interaction between CCDC103 and SPAG6 in the presence of DMSO, nocodazole and paclitaxel. (D) Schematic of WT CCDC103 protein and three mutations found in PCD patient mutations. Relative severity of the patient PCD phenotype associated with the mutation indicated in parentheses. (E) BRET ratios for the interaction between DYNC1H1 and WT and mutant CCDC103 proteins. (F) BRET ratios for the interaction between SPAG6 and WT and mutant CCDC103 proteins. **P<0.005, *P<0.05; ns, not significant (two-tailed unpaired Student's *t*-test).

Based on biochemical evidence showing that CCDC103 forms self-organizing oligomers, others have hypothesized that CCDC103 may function as a molecular scaffold, anchoring other proteins at the microtubule and facilitating their function (King and Patel-King, 2020). However, very little is known about the network of CCDC103-interacting proteins. To elucidate conserved CCDC103-interacting proteins, which could provide mechanistic insights into how CCDC103 regulates microtubule stability within the cytoplasm of myeloid cells, we performed a yeast two-hybrid screen using the zebrafish Ccdc103 protein against a normalized human cDNA library. Interestingly, two of the interacting peptide sequences identified in this screen included the C-termini of DYNC1H1, which validated the previous colocalization of CCDC103 and DYNC1H1 on microtubules that we observed with IHC (Fig. 2), and SPAG6 (Fig. 7A). The interaction with SPAG6 was particularly intriguing due to a number of phenotypic and functional similarities to CCDC103: mouse *Spag6* mutants display ciliary defects and many phenotypic hallmarks of PCD (Sapiro et al., 2002); Spag6 can promote effective migration of cortical neurons and proliferation and migration of mouse embryonic fibroblasts (Sapiro et al., 2002; Li et al., 2015; Alciaturi et al., 2019); Spag6 has increased expression in myeloid leukemia lines (Cooley et al., 2016; Yang et al., 2015; Yin et al., 2018); and Spag6 can promote microtubule stability (Zheng et al., 2019). To validate these protein–protein interactions, we used the bioluminescent resonance energy transfer (BRET) assay-based LuTHy system (Trepte et al., 2018). The BRET assay confirmed that CCDC103 interacts with both the C-terminal portion of DYNC1H1 (Fig. 7B) and the full-length SPAG6 (Fig. 7C). To determine whether this interaction required the presence of intact microtubules, we treated transfected cells with nocodazole and saw that it significantly abrogated the interactions between CCDC103 and both DYNC1H1 and SPAG6 (Fig. 7B,C), whereas paclitaxel treatment did not affect the strength of the measured interactions.

Currently, there are three known alleles in *CCDC103* that can cause PCD in humans, which result in highly variable clinical presentations (Fig. 7D) (Panizzi et al., 2012). To determine whether these alleles affect the ability of CCDC103 to interact with DYNC1H1 and SPAG6, we performed the BRET assays with the *CCDC103* mutant alleles. Interestingly, the predicted severity of the mutations in CCDC103, which correlate with the overt severity of the patient PCD phenotypes, also correlated with the loss of avidity in the interaction between CCDC103 and DYNC1H1 or SPAG6 (Fig. 7E,F) (Panizzi et al., 2012). Interestingly, the most severe mutation, CCDC103^G128fs*^, disrupted interactions with DYNC1H1 and SPAG6, similar to what was found with the nocodazole treatments (Fig. 7B,C). Furthermore, although both DYNC1H1 and SPAG6 showed similar trends with respect to the progressive loss of interaction due to the different CCDC103 mutations, the interactions between SPAG6 and CCDC103 appeared to be more sensitive to these mutations than DYNC1H1, as indicated by a significant impairment of interaction relative to control with both the CCDC103^H154P^ and CCDC103^G128fs*^ mutations. Taken together, these results signify that patient mutations in *CCDC103* that underlie PCD disrupt microtubule-dependent interactions between CCDC103 and both DYNC1H1 and SPAG6.

### Loss of SPAG6 recapitulates *smh* mutant myeloid defects

**Fig. 8.**
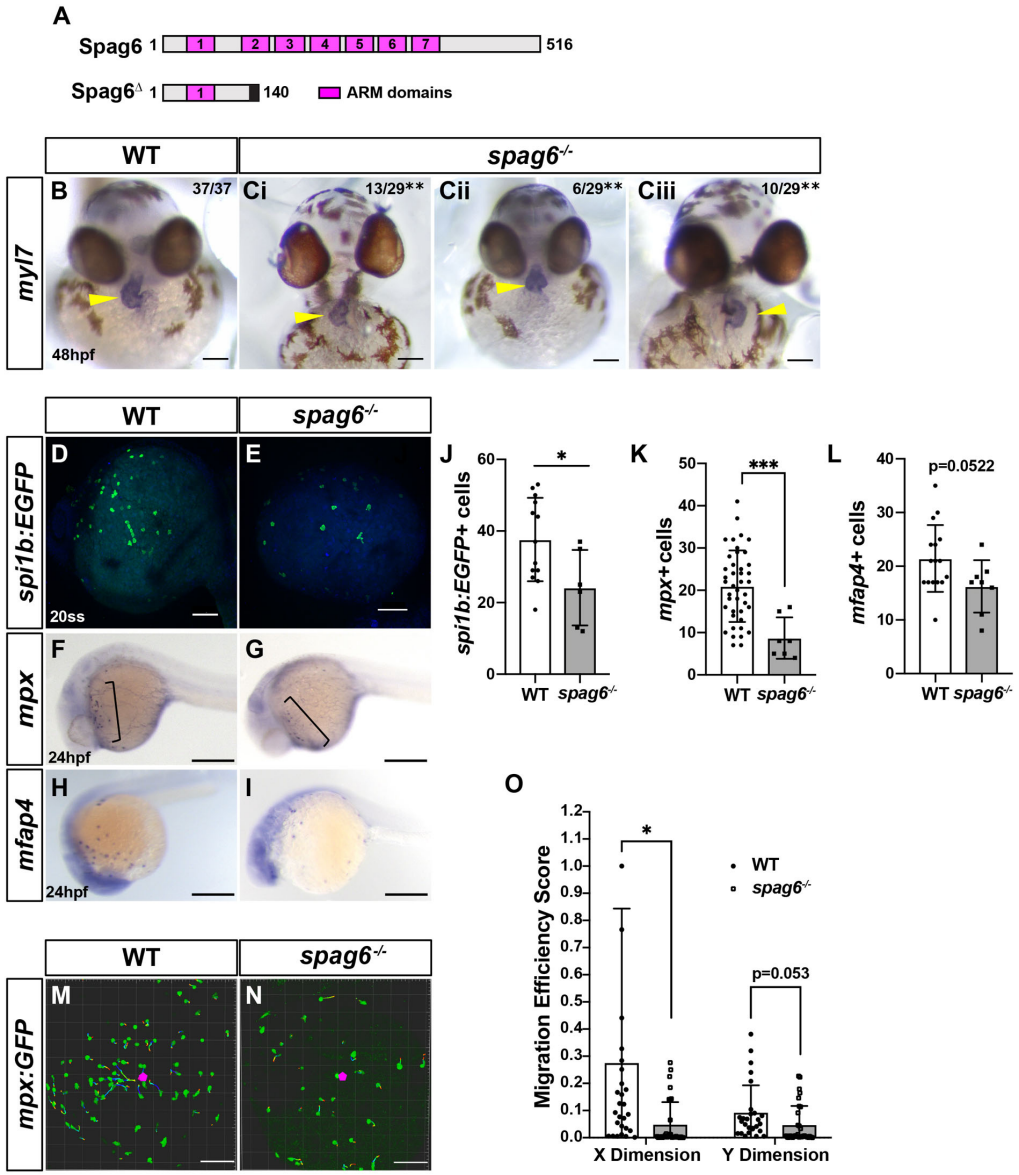
**Spag6 is required for normal myeloid proliferation and migration.** (A) Domain architecture for WT zebrafish Spag6 and the predicted truncation from the *spag6* mutant allele used*.* (B,Ci-iii) ISH for *myl7* in WT and *spag6* mutant embryos at 48 hpf. (B,Ci) Normally (situs solitus, dextral) looped hearts in WT and *spag6* mutant embryos. (Cii) Linearized heart in *spag6* mutant. (Ciii) Reverse (situs inversus) heart in *spag6* mutant. Yellow arrowheads indicate hearts. Fractions indicate the number of embryos with the given phenotype. **P<0.001 (Fisher's exact test). (D,E) Whole-mount IHC for *spi1b:EGFP* in WT (*n*=11) and *spag6* mutants (*n*=8). (F,G) Whole-mount ISH for the neutrophil marker *mpx* in WT (*n*=45) and *spag6* mutant (*n*=11) embryos at 24 hpf. (H,I) Whole-mount ISH for the macrophage marker *mfap4* in WT (*n*=19) and *spag6* mutant (*n*=8) embryos at 24 hpf. (J-L) Quantification of myeloid progenitors (*spi1b:EGFP^+^*), neutrophils (*mpx^+^*) and macrophages (*mfap4^+^*) from a single yolk hemisphere of the individual embryos. (M,N) Representative confocal projection images from wounding experiments of WT sibling (*n*=4) and *spag6^−/−^* (*n*=4) *mpx:EGFP* transgenic embryos at 24 hpf. (O) Migration efficiency scores from cell tracks of yolk wounding assays in WT and *spag6* mutant *mpx:EGFP^+^* embryos at 24 hpf. ***P<0.0001, *P<0.05 (two-tailed unpaired Student's *t*-test). Scale bars: 50 μm (B-Ciii), 300 μm (F-I), 100 μm (D,E,M,N).

In light of reports implicating SPAG6 in myeloid proliferation and cell migration (Sapiro et al., 2002; Jiang et al., 2019; Zheng et al., 2019), we interrogated the requirement of Spag6 in zebrafish myeloid cells. RT-PCR from flow-sorted *spi1b:EGFP* cells showed that zebrafish *spag6* is expressed in myeloid cells (Fig. S8). Zebrafish *spag6* mutants that harbor a 44 bp deletion, which is predicted to cause a truncation via introduction of a premature stop codon in the second Armadillo repeat domain, were generated using CRISPR/Cas9 (Fig. 8A; Fig. S8). In addition to generating a truncated protein, RT-qPCR showed that the *spag6*^Δ*44*^ transcript in this allele likely underwent non-sense mediated decay (Fig. S8). Fish carrying this *spag6* allele were homozygous viable and did not show dramatic overt signs of PCD that are observed in *smh* or other PCD zebrafish models, such as ventral curving of the body axis or pronephric cysts (Fig. S8) (Panizzi et al., 2012; Zariwala et al., 2013; Omori and Malicki, 2006; Hjeij et al., 2013). However, *spag6* homozygous males were largely infertile, with fertilization rates of ∼13% (nine embryos fertilized out of 72 from one representative experiment) when crossed, consistent with observations of male factor infertility in *Spag6* mutant mice (Sapiro et al., 2002). Despite the lack of body curvature and pronephric defects in *spag6* mutants, we observed that *spag6* mutants generated from crosses of *spag6^+/−^* females and *spag6^−/−^* males exhibited laterality defects, including situs inversus and linearized hearts (Fig. 8B,C), suggesting that they have hypomorphic motile cilia defects. With respect to myeloid cells, we found that *spag6* mutants have fewer myeloid progenitors (*spi1b:EGFP^+^*) (Fig. 8D,E,J), neutrophils (*mpx+*) (Fig. 8F,G,K) and macrophages (*mfap4^+^*) (Fig. 8H,I,L). Furthermore, neutrophils and macrophages in *spag6* mutants had decreased directed migration efficiency and more rounded morphology (Fig. 8M-O; Movies 9 and 10, Fig. S9). Thus, our data support that Spag6 regulates myeloid development and function in a similar manner to Ccdc103 that is consistent with promoting microtubule stability.

## DISCUSSION

Our data provide novel insights into cytoplasmic, extraciliary functions of CCDC103, in which mutations underlie a significant percentage of currently identified PCD cases (Shoemark et al., 2018). Importantly, our data suggest that CCDC103 is required to promote normal proliferation and migration of myeloid cells through enhancing cytoplasmic microtubule stability. Previous biochemical studies have shown that CCDC103 forms self-organizing oligomers, and binds directly to and stabilizes cytoplasmic microtubules assembled in solution (King and Patel-King, 2015). Consequently, it was speculated that CCDC103 might serve as a scaffolding or adaptor protein, which could facilitate the function of multiple proteins that require localization at the microtubule. However, these proposed functions were not verified *in vivo* and it was unclear how they related to roles in motile cilia. Our data corroborate the association of CCDC103 with microtubules *in vivo* and support that these interactions occur within the cytoplasm of myeloid cells. Intriguingly, although interactions between CCDC103 and axonemal dyneins have been demonstrated, we show that, within the cytoplasm, CCDC103 has microtubule-dependent interactions with cytoplasmic dynein (DYNC1H1). Thus, our results imply that one requirement of CCDC103 independent of its functions in motile cilia may be to stabilize microtubule–dynein interactions that regulate dynein-dependent cellular processes within the cytoplasm, including cargo transport and nuclear positioning (Vallee et al., 2012; Roberts et al., 2013).

With the exceptions of axonemal dynein and microtubules, CCDC103-dependent protein complexes have not been identified (Panizzi et al., 2012). Accordingly, in addition to DYNC1H1, we identified SPAG6 as a novel interactor of CCDC103. One reason it was an attractive candidate is because of the large body of literature indicating that Spag6 promotes the same cellular processes as Ccdc103 in both ciliated and non-ciliated cells. Broadly, Spag6 has been shown to regulate cilia assembly, microtubule stability, proliferation and migration (Li et al., 2015; Zheng et al., 2019). Although mutations in *SPAG6* have not as yet been identified as underlying cases of PCD in humans, *Spag6* KO mice have a higher incidence of neonatal death, with ∼50% showing hydrocephalus (Sapiro et al., 2002). Furthermore, *Spag6* KO mice that survive have cilia defects in the tracheal epithelia and columnar cells of the middle ear, and the males are infertile (Sapiro et al., 2002). Like *ccdc103*, in zebrafish, *spag6* is also expressed in the pronephros and spinal cord and is responsive to FoxJ1 (also known as Foxj1a), a master regulator of the motile cilia program (Vij et al., 2012). Furthermore, a screen in zebrafish for genes that regulate motile cilia showed that morpholino oligo-mediated depletion of *spag6* resulted in pronephric cysts, but not other characteristics of PCD-associated defects in zebrafish, such as body curvature and hydrocephalus (Austin-Tse et al., 2013). Although the *spag6* mutant allele we generated showed randomization of heart laterality, which is indicative of motile cilia impairment, the mutants were viable and otherwise did not result in additional overt PCD-related defects. However, similar to *Spag6* KO mice, the homozygous *spag6* zebrafish males were largely infertile. In mice, there is functional redundancy of Spag6 with Spag6-like (Spag6L) and *Spag6*; *Spag6l* KO mice have a higher frequency of early embryonic lethality and hydrocephalus (Cooley et al., 2016). However, zebrafish do not have a *Spag6l* ortholog (zfin.org). Hence, the lack of other more severe overt phenotypes consistent with motile cilia defects could be due to redundancy with other Spag genes. Although it has not been confirmed with zebrafish mutants, *spag1a* has been implicated in PCD in zebrafish (Knowles et al., 2013) as mutations in SPAG1 underlie PCD in humans (Knowles et al., 2013). Furthermore, we observe non-sense mediated decay with our *spag6* allele, which can trigger increased expression of even distantly related genes, leading to genetic compensation and minimization of defects (Rossi et al., 2015).

In addition to the correlations in ciliated cells within the literature, our data support that zebrafish *smh* and *spag6* mutants both have previously unrecognized defects in myeloid proliferation and directed migration, phenotypes that are consistent with decreased microtubule stability. Increased SPAG6 expression is found in a broad range of myeloid cancers and myelodysplastic syndromes (Zheng et al., 2019; Steinbach et al., 2015; Yin et al., 2018). Depletion of SPAG6 is associated with reduced proliferation of these leukemia lines, implying that its increased expression contributes to their hyperplasia. Interestingly, it has also been suggested that SPAG6 promotes microtubule stability and longevity through promoting microtubule acetylation (Li et al., 2015), although the specific mechanisms by which this happens are not currently understood. While CCDC103 may be sufficient to increase microtubule stability independent of other factors *in vitro* (King and Patel-King, 2015), it is feasible that SPAG6-mediated microtubule acetylation may contribute to the microtubule-stabilizing effects of CCDC103 *in vivo*. Moreover, it is interesting that CCDC103–SPAG6 interactions are more sensitive to the patient PCD-associated CCDC103 mutants than DYNC1H1, which implies that the failure of these proteins to complex may be a key etiology driving both canonical PCD-associated defects in tissues with motile cilia and myeloid defects independent of motile cilia. Overall, we propose that CCDC103 may anchor the interactions of SPAG6 with microtubules to enhance their stability in the cytoplasm of myeloid cells, as well as within the axoneme of cells with motile cilia.

We currently do not understand whether enhancement of microtubule stabilization in myeloid cells is a general requirement of canonical PCD-associated proteins or is unique to CCDC103. Given that mutations in CCDC103 only account for a small number of known PCD cases (Knowles et al., 2013; Horani et al., 2016), we postulate that other PCD-associated proteins will have requirements promoting the production and function of myeloid cells through enhancing microtubule stability. All dynein axonemal assembly factors are also localized within the cytoplasm, supporting that they have additional molecular functions in other cell types similar to Ccdc103. For example, dyslexia susceptibility 1 candidate 1 [DYX1C1; also called dynein axonemal assembly factor 4 (DNAAF4)] is critical for motile ciliogenesis and axonemal dynein assembly and is intimately involved in neuronal migration (Casey et al., 2015; Tarkar et al., 2013; Chandrasekar et al., 2013). Furthermore, DYX1C1 has been shown to associate with cytoskeletal proteins, which may contribute to its effects on cellular migration (Tammimies et al., 2013). It has also been implicated in the proliferation of certain breast cancer cell subtypes because of its ability to modulate the function of the estrogen receptor (Massinen et al., 2009).

In conclusion, our study provides mechanistic insight into largely overlooked chemotactic defects found in neutrophils from PCD patients. Much remains to be learned about the pathophysiology of PCD. In particular, we envision that our observations may be relevant to the etiology of persistent pulmonary infections in these patients. Moving forward, deciphering the precise molecular mechanism by which CCD103–SPAG6 complexes promote microtubule stability and whether there are broad requirements of PCD-associated proteins in facilitating myeloid proliferation and migration will enhance our understanding of immunological deficiency that may contribute to PCD, which could open avenues into novel treatment modalities to improve outcomes for these patients.

## MATERIALS AND METHODS

### Ethics statement

All zebrafish husbandry and experiments were performed as described in approved Institutional Animal Care and Use Committee (IACUC) protocols at the Cincinnati Children's Hospital Medical Center.

### Zebrafish husbandry, transgenic and mutant lines

Adult zebrafish (*Danio rerio*) were raised and maintained under standard laboratory conditions (Westerfield, 2000). Zebrafish lines used were *spi1b:EGFP^gl21Tg^* (Ward et al., 2003), *mpeg1.1:YFP^w200Tg^* (Roca and Ramakrishnan, 2013), *mpx:GFP^uwm1Tg^* (Mathias et al., 2006) and *smh^tn222a^* (Panizzi et al., 2012). *smh* mutants were genotyped according to the protocols as previously described and with the primers listed (Table S1).

Zebrafish *spag6* mutants were created with standard CRISPR/Cas9 methods. Two guide RNAs (gRNAs) (Table S1) to exon 4 of *spag6* were injected with 150 pg each along with Cas9 protein (NEB, M0646M). Injected embryos were screened for efficacy in creating deletions with PCR. The remaining F0 embryos were raised and subsequently screened for germline deletions in *spag6*. A *spag6* allele with a 44 bp deletion was used that is predicted to cause the protein to go out of frame after AA 131 and include a nine-AA extension prior to a stop codon. The allele used is designated *spag6^ci1013^*.

### mRNA injections

Zebrafish embryos were injected at the one-cell stage with the following: 600 pg *ccdc103* mRNA, 150 pg each of *scl* and *lmo2* mRNA, and 100 pg *spi1b* mRNA. Capped mRNA was made using a Message Machine Kit (Ambion) according to the manufacturer's instructions.

### Whole-mount ISH

ISH was performed as reported previously (D'Aniello et al., 2013). Probes for the following genes were used: *mpx* (ZDB-GENE-030131-9460), *mfap4* (ZDB-GENE-040426-2246), *myl7* (ZDB-GENE-991019-3) and *ccdc103* (ZDB-GENE-040718-253). Embryos were visualized and photographed using a Zeiss M2BioV12 Stereo microscope.

### Directed migration and cell morphology analysis

*smh* and *spag6* embryos bearing either the *mpeg1.1:YFP* or *mpx:GFP* transgene were anaesthetized using ethyl 3-aminobenzoate methanesulfonate (tricaine) at a standard concentration of 164 mg/l (Sigma-Aldrich) and mounted in 0.6% low-melt agarose dissolved in embryo medium. Wounds on the yolk were made in 24 hpf embryos with an upright Nikon FN1 microscope on an A1R confocal scanner. The scan area was decreased by 500× zoom, and the shutter was opened for 5 s. Wound sites were able to be visualized by autofluorescence in the GFP channel. Time-lapse images were captured with a 16× water immersion objective at 1.5× zoom. For each embryo, 150 μM *z*-stacks were collected at 5 min intervals for 1 h. Cell sphericity (calculated from *z*-stacks), track speed and positional data were all captured and analyzed in Imaris (Bitplane).

Migration efficiency was calculated using Imaris by determining each individual cell's motion vector in both the X and Y dimensions. These data were compared to a unique ‘ideal’ vector for each cell, which represented the path that a given cell would need to take to reach the wound site by the conclusion of the time lapse. Because the average distance in the Z dimension between a given cell and the wound site was only ∼10-15 μM, migration efficiency in that dimension was not calculated.

For imaging single cells, live wounded or unwounded embryos at either 24 hpf or 28 hpf (as specified) were imaged on a Nikon A1 using a 60× water-immersion objective at 5× zoom for the time-lapse duration and at the intervals specified. Cell circularity, which was calculated from the single image planes, was calculated and analyzed in Imaris (Bitplane). When indicated, embryos were treated with 1 nM paclitaxel (Sigma-Aldrich) solubilized in DMSO for 45 min prior to wounding and EdU assays. Experiments were then carried out as described above.

### RT-qPCR

Total RNA isolation and RT-qPCR was performed using previously reported methods (D'Aniello et al., 2013). Total RNA was isolated from staged embryos or FACS-sorted *spi1b:EGFP*^+^ myeloid progenitors that were homogenized in TRIzol (Ambion) and collected using RNeasy mini columns (Qiagen). The TURBO DNA-free kit (Applied Biosystems) was used to remove genomic contamination. One microgram of RNA was used for cDNA synthesis using a ThermoScript Reverse Transcriptase kit (Invitrogen). Quantitative PCR was performed using standard PCR conditions in a Bio-RadCFX PCR machine with Power SYBR Green PCR Master Mix (Applied Biosystems). Expression levels were standardized to β-actin or *GAPDH* expression. All experiments were performed in triplicate. cDNA for all myeloid cell lines, CD34^+^CD38^–^ HSCs and whole cord blood was a gift from Dr Jim Malloy (Cincinnati Children's Hospital Medical Center, Cincinnati, OH, USA).

### EdU assay

EdU labeling was carried out using a Click-iT EdU Alexa Fluor Imaging Kit (Molecular Probes) according to the manufacturer's instructions. Briefly, dechorionated embryos at the 20-ss were incubated with 10 mM EdU for 30 min on ice, the EdU was washed out, and embryos were incubated at 28.5°C until 24 hpf. Embryos were then fixed overnight in 4% paraformaldehyde (PFA) in PBS at 24 hpf and the Click-iT reaction was performed according to the manufacturer's protocol. After EdU incorporation, embryos were processed for IHC using chicken anti-GFP antibody (1:250) and 4′,6-diamidino-2-phenylindole (DAPI; 1:5000). Embryos were imaged using a Nikon A1 confocal microscope and analyzed with Imaris (Bitplane).

### HL-60 cell culture and differentiation

HL-60 cells were maintained in Iscove's modified Dulbecco's medium (IMDM) supplemented with 20% bovine growth serum (BGS) as per American Type Culture Collection (ATCC) instructions. Cells were differentiated to neutrophils according to established methods (Tasseff et al., 2017). In short, neutrophil differentiation was performed by culturing for 96 h in 1 µM all*-*trans retinoic acid (ATRA) in an eight-well chamber slide (Ibidi) or in suspension. HL-60 cells were differentiated into macrophage-like cells using 200 ng/ml phorbol 12-myristate 13-acetate (PMA) for 48 h, in coated chamber slides or in suspension, according to established methods.

### Protein–protein interaction analysis

Ccdc103 was screened for candidate interacting proteins using the Matchmaker Gold Yeast Two-Hybrid System (Takara, 630489) as per the manufacturer's instructions. Full-length zebrafish *ccdc103* cDNA was used as bait and screened against a Mate & Plate – Universal Human Library (Normalized) (Takara, 630481). Then, 128 positive colonies were sequenced representing 66 different cDNAs. Candidate proteins were prioritized for additional analysis based on expression and known functions.

BRET analysis of CCDC103–DYNC1H1 and CCDC103–SPAG6 interactions was carried out as described in Trepte et al. (2018). HEK293T cells were plated at a density of 1×10^–5^ cells/ml in a 96-well plate and co-transfected with full-length CCDC103 coding sequence (CDS) fused to a Nano-luciferase (NL)-Myc tag (Addgene plasmid #113447) and either the full CDS (SPAG6) or, in the case of DYNC1H1, a 1000-AA length of the C-terminal portion fused to a proteinA-mCitrine tag (Addgene plasmid #113449). FuGENE^®^ 6 Transfection Reagent (Promega) was used for all transfections. Forty-eight hours after transfection, Coelenterazine-h (Sigma-Aldrich, C3230) was added to a final concentration of 5 µM. Plates were incubated at 37°C for 15 min and then read on a Synergy H1 microplate reader (Biotek). Short-wavelength (370-480 nM) and long-wavelength (520-570 nM) luminescence values were collected on spectral scanning mode, with an integration time of 1000 ms.

### IHC

For IHC of differentiated and undifferentiated HL-60 cells, suspension cultures or chambered coverslips, the following protocol was used. First, growth medium was aspirated from either coverslips or pelleted cells. Cells were washed 3× in room temperature PBS and fixed for 1 h at 25°C in 4% PFA in PBS. Cells were washed 3× and then permeabilized in PBS containing 0.1% Tween 20/0.1% Triton X-100/1% DMSO for 15 min. Cells were washed again and then incubated with the specified primary antibodies (Table S2) for 1 h at room temperature in a block solution [1% bovine serum albumin (BSA), 1% DMSO, 0.1% Tween 20 in PBS], washed and incubated for 1 h in secondary antibody+blocking solution. Cells were protected from light. Following all washes, cells were mounted in SlowFade™ Diamond Antifade Mountant with DAPI (Thermo Fisher Scientific).

For all whole-mount IHC, all of the above procedures were followed, except embryos were fixed at the developmental stages specified in 4% PFA in PBS overnight at 4°C or for 2 h at 25°C (overnight), and all antibody incubations were performed overnight at 4°C. The affinity-purified rabbit polyclonal pan-CCDC103 antibody was generated by YenZym Antibodies (www.yenzym.com) through a combination immunization approach with peptides for AAs 54-74 (SHLKPLEQKDKMGGKRFVPWN) of mouse Ccdc103 and AAs 54-74 (SHLKPLDRNDISGSPRKQPWN) of zebrafish Ccdc103. The antibody was verified via comparison to a previously available commercial antibody (Abcam, ab177558), whole-mount IHC, which showed a lack of expression in *smh* mutants, and western blotting (Fig. S3).

All imaging was performed on a Nikon Eclipse Ti inverted microscope on a Nikon A1R confocal and processed in NIS-Elements AR or Imaris, as appropriate. Images were processed using Nikon denoise.ai functionality.

### Western immunoblotting

Low-passage HEK293T cells, maintained in Dulbecco's modified Eagle medium (DMEM)+10% BGS+1% penicillin/streptomycin, were transfected with either control plasmid or human pCS2P-CCDC103-myc (gift from Iain A. Drummond, MDI Biological Laboratory, Bar Harbor, ME, USA) for 24 h. Samples were lysed in RIPA buffer (10 mM Tris-HCl, pH 8.0, 1 mM EDTA, 1% Triton X-100, 0.1% sodium deoxycholate, 0.1% SDS, 140 mM NaCl) and incubated at 95°C for 5 min. A volume of sample corresponding to 30 μg of total protein was separated by electrophoresis on a 4-20% gradient Tris-glycine gel (Bio-Rad) and transferred to nitrocellulose membrane, blocked in 5% BSA in Tris-buffered saline with 0.1% Tween (TBST) and incubated overnight at 4°C with 1:500 rabbit anti-CCDC103 antibody (YenZym Antibodies) and 1:2000 mouse anti-tubulin antibody (Sigma-Aldrich, T6199). Membranes were washed and incubated for 1 h in 1:15,000 goat anti-rabbit IRDye 680CW (LICOR) and 1:15,000 goat anti-mouse IRDye 800Rd (LICOR), and bands were visualized on the LICOR Odyssey imaging system.

### Statistical analysis

Unless otherwise specified, all statistical tests were carried out using two-tailed unpaired Student's *t*-test with an α value of *P*<0.05. For cardiac laterality experiments, significance was determined by Fisher's exact test. Sphericity and circularity indices were determined by indwelling algorithms in Imaris (Bitplane). All statistical analyses were performed in GraphPad Prism 6.0. All data presented in this paper are given as the pooled results of at least three independent experiments with embryos gathered from independent crosses.

## Supplementary Material

Supplementary information
