# Supplementary material for: Ccdc103 promotes myeloid cell proliferation and migration independent of motile cilia
Source: Dis Model Mech. 2021 May 24;14(5):dmm048439. doi: 10.1242/dmm.048439 (PMC8214733; doi:10.1242/dmm.048439)
Supplement: Supplementary information [file dmm-14-048439-s1.pdf]

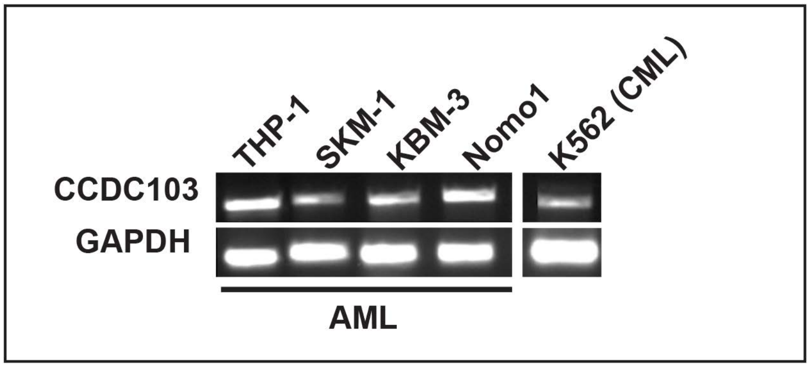

Figure S1. CCDC103 is expressed in multiple human cell lines of myeloid origin. RT-PCR for *CCDC103* performed on cDNA from a panel of myeloid leukemia cell lines.

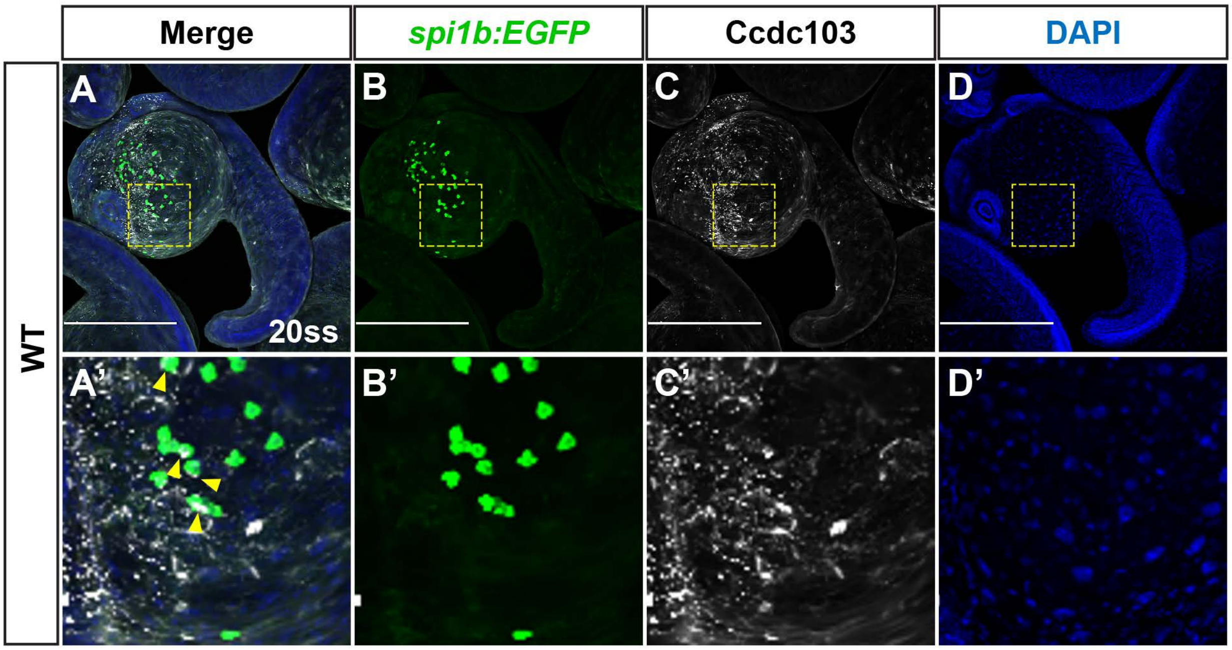

Figure S2. Zebrafish Ccdc103 is expressed in differentiated myeloid cells.
(A-D') IHC images from CCDC103-stained *spi1b:EGFP*+ transgenic embryos at the 20ss, showing Ccdc103 staining and co-localization of Ccdc103 and GFP. Yellow arrowheads - regions of *spi1b:GFP*+ cells with Ccdc103. Scale bars: 500 μm.

Disease Models & Mechanisms • Supplementary information

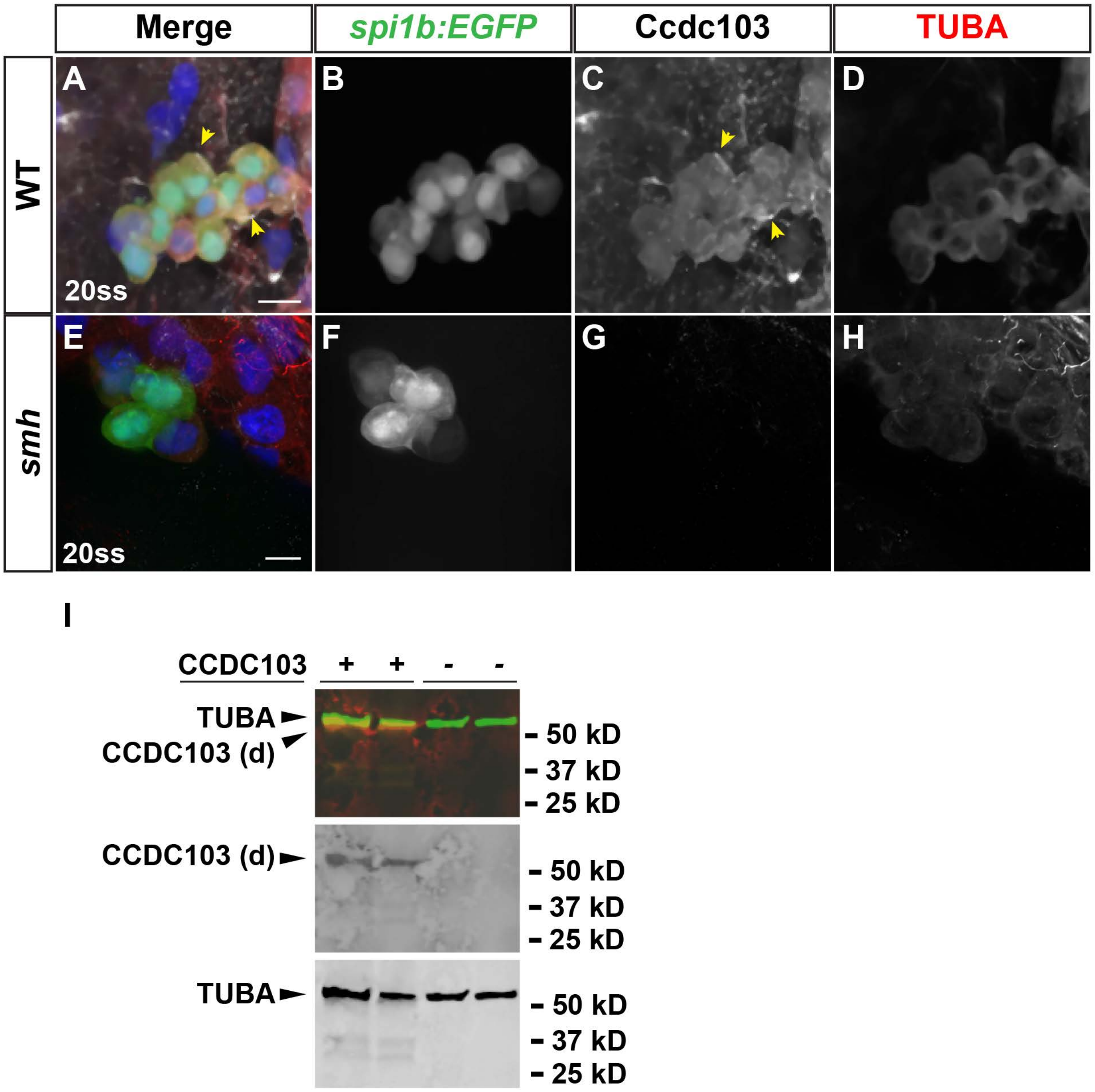

Figure S3. Ccdc103 expression is lost in zebrafish *smh* mutants.
(A-H) Whole-mount IHC for Ccdc103 in WT and *smh* mutant *spi1b:EGFP* embryos at 20ss. Ccdc103 was not detected in *smh* mutants. Arrowheads indicated Ccdc103. Scale bars: 10 µm. (I) Western blot of HEK293 cells transfected with human CCDC103. The custom CCDC103 antibody predominantly recognizes dimers (d) of CCDC103 at ~54kD. The predicted molecular weight of CCDC103 monomers is ~27 kD. Detection of CCDC103 dimers even under strong reducing conditions is consistent with what was reported previously by Panizzi et al. (2012). The size of TUBA is ~55kD.

Disease Models & Mechanisms • Supplementary information

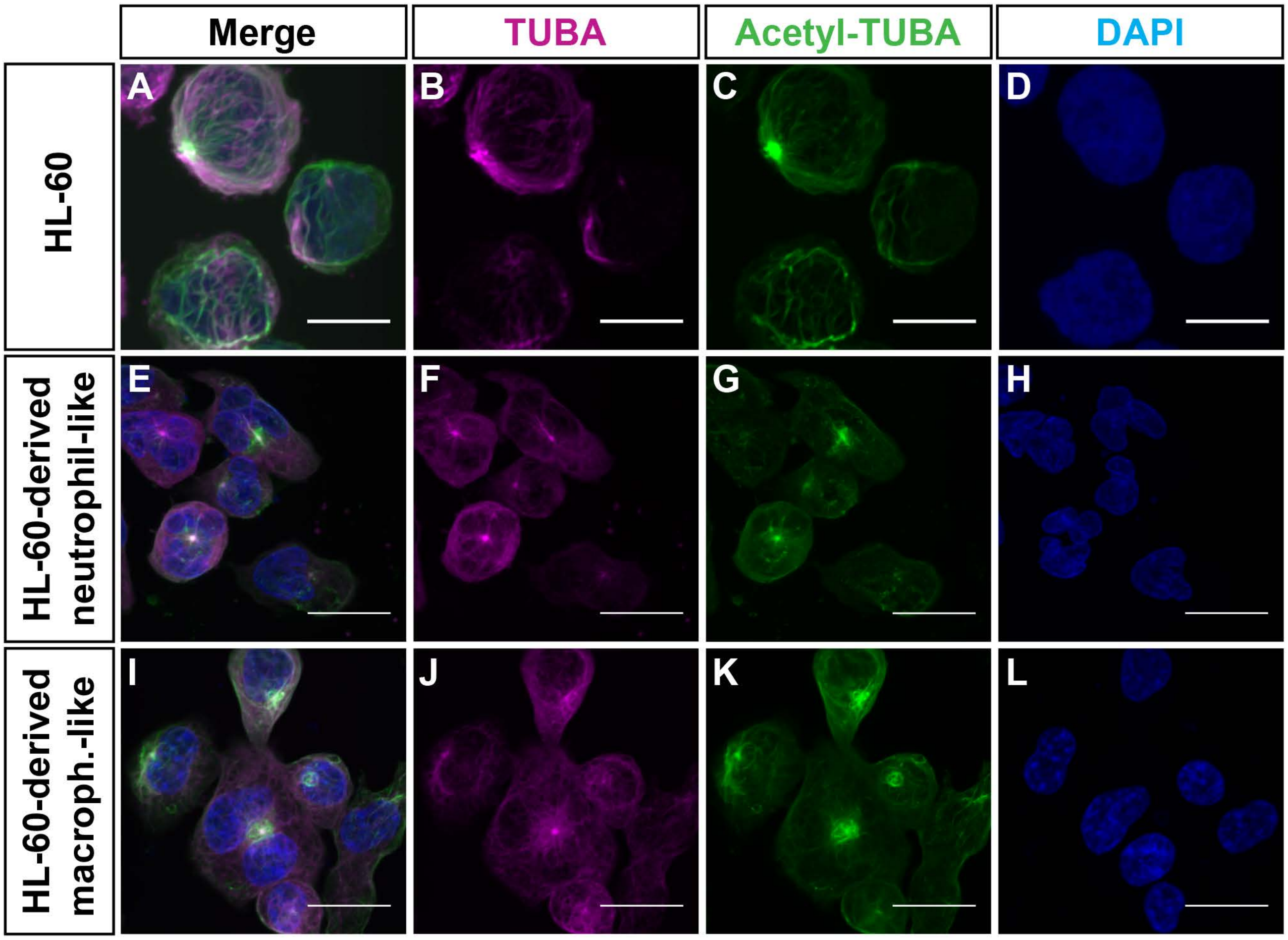

Figure S4. Acetylated TUBA staining of undifferentiated and differentiated HL-60 cells. (A-L) IHC for TUBA and acetylated (K40) TUBA in undifferentiated HL-60 cells, neutrophil-like and macrophage-like cells differentiated from HL-60 cells. Scale bars: 10 μm.

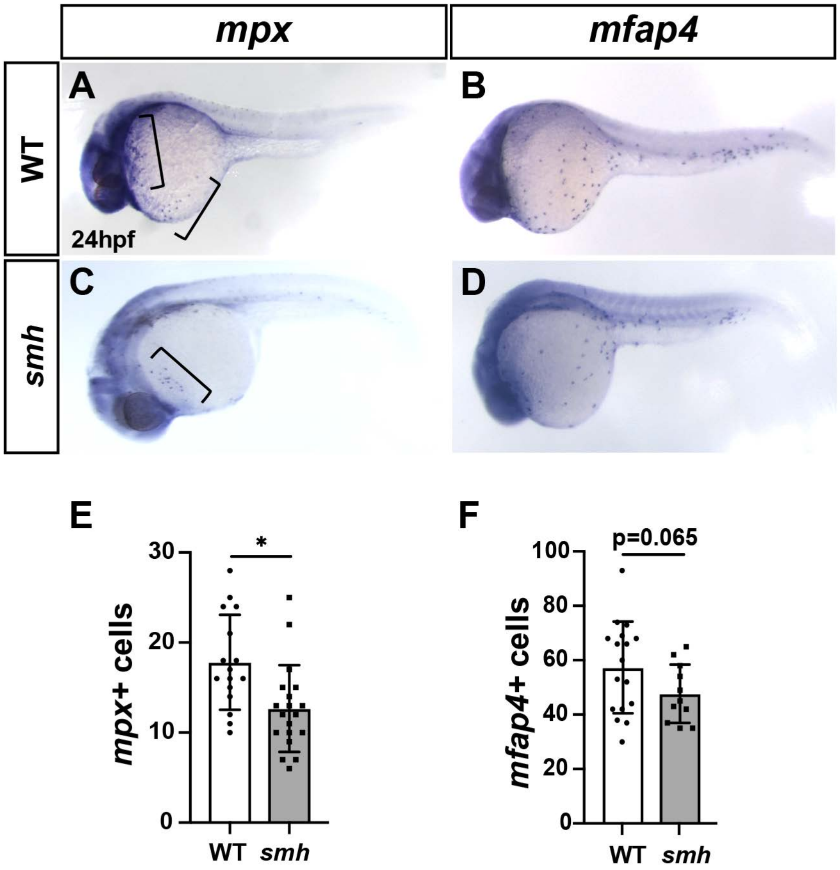

Figure S5. *Smh* mutant embryos have fewer neutrophils and macrophages.
(A,B) Whole-mount ISH in WT (n=16) and *smh* (n=19) zebrafish embryos for the neutrophil marker *mpx*. Clusters of *mpx*+ cells are indicated by brackets. (C,D) Whole-mount ISH in WT (n=17)and *smh* (n=11) zebrafish embryos for the macrophage marker *mfap4.* (E) Quantification of the number of *mpx*+ cells on one hemisphere of the yolk from individual embryos. For E, * - $p<0.05$. (F) Quantification of the number of *mfap4*+ cells on one hemisphere of the yolk from individual embryos.

Disease Models & Mechanisms • Supplementary information

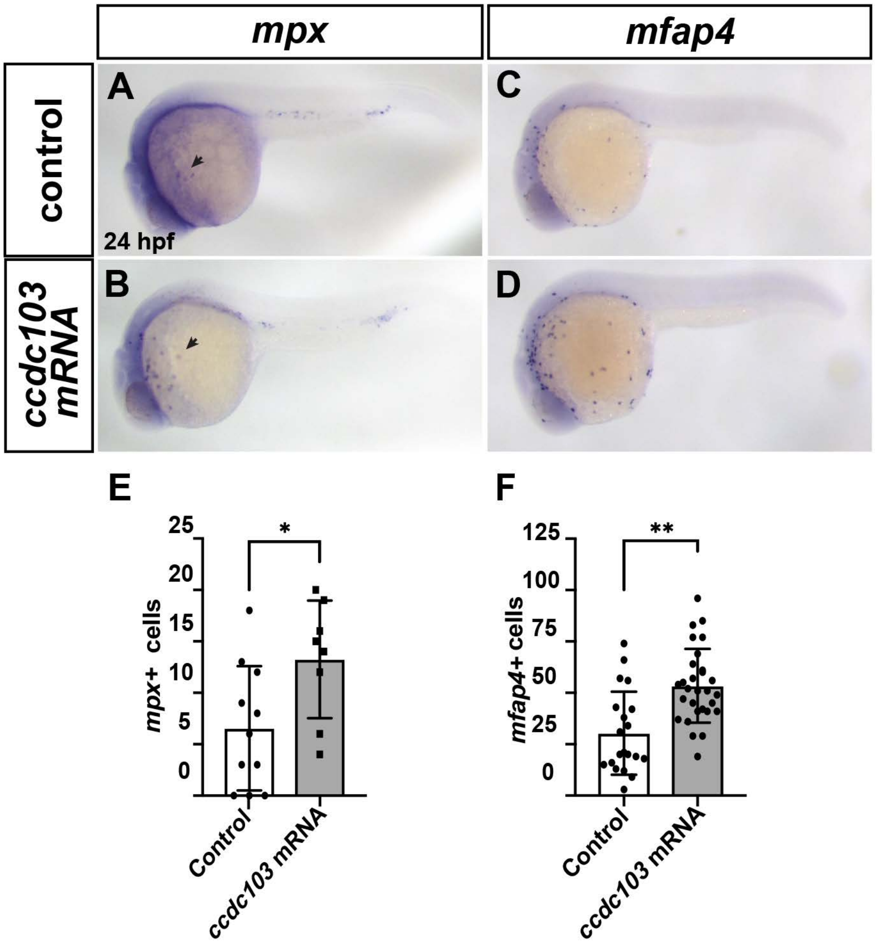

Figure S6. *Ccdc103* mRNA injection increases neutrophils and macrophages.
(A,B) Whole-mount ISH in control (n=11) and *ccdc103* mRNA-injected (n=8) zebrafish embryos for neutrophil marker *mpx*. Clusters of *mpx*+ cells are indicated by black arrow. (C,D) Whole-mount ISH in controls (n=19) and *ccdc103* mRNA-injected (n=29) zebrafish embryos for the macrophage marker *mfap4*. (E) Quantification of the number of *mpx*+ cells on one hemisphere of the yolk from individual embryos. (F) Quantification of the number of *mfap4*+ cells on one hemisphere of the yolk from individual embryos. For E and F, ** - $p<0.005$, * - $p<0.05$.

Disease Models & Mechanisms • Supplementary information

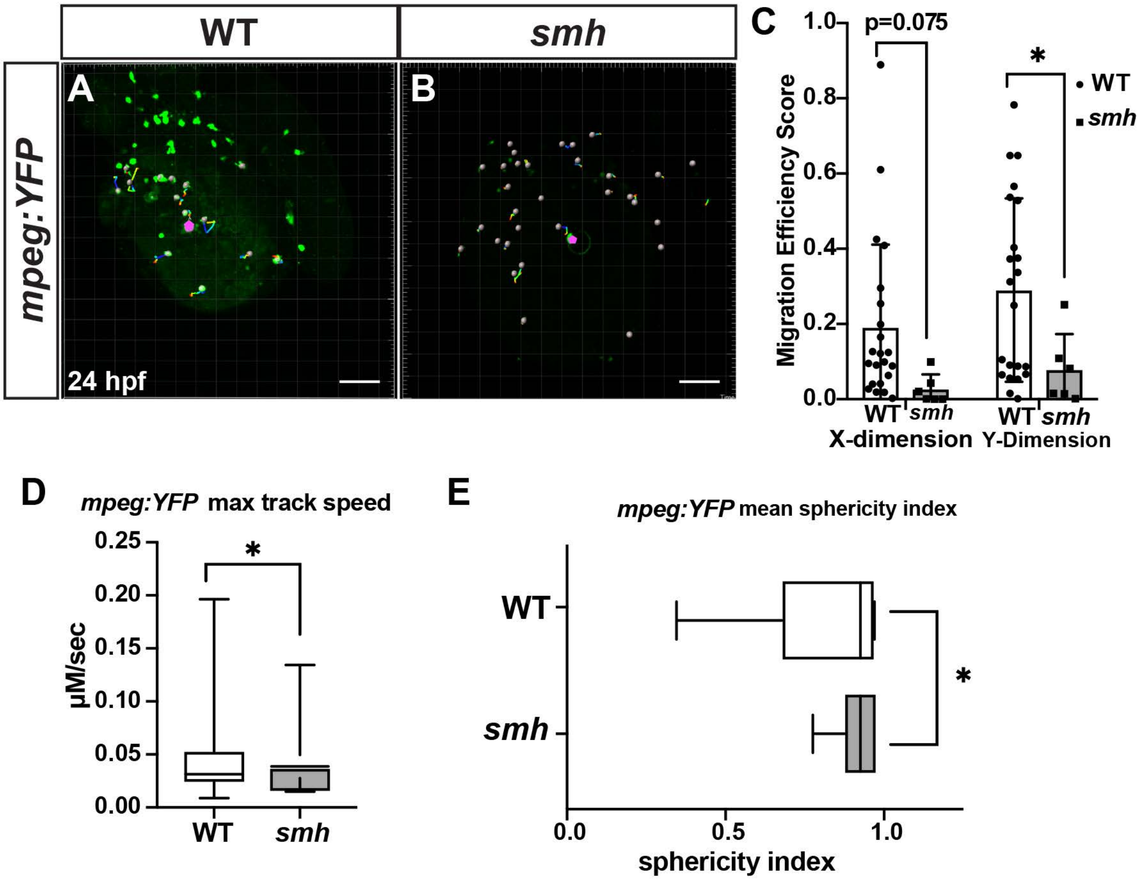

Figure S7. *Smh* mutant macrophages display directed migration defects in response to sterile yolk wounds.
(A,B) Cell tracks for WT and *smh* mutant *mpeg:YFP* embryos. (C) Quantification of migration efficiency scores calculated from point position data generated in Imaris. Each data point represents an individual cell from a minimum of 3 separate experiments, per genotype. (D) Quantification of max track speed. (E) Mean cell sphericity indices as calculated in Imaris. For C, D, and E, * - $p<0.05$. Scale bars: 100 μm.

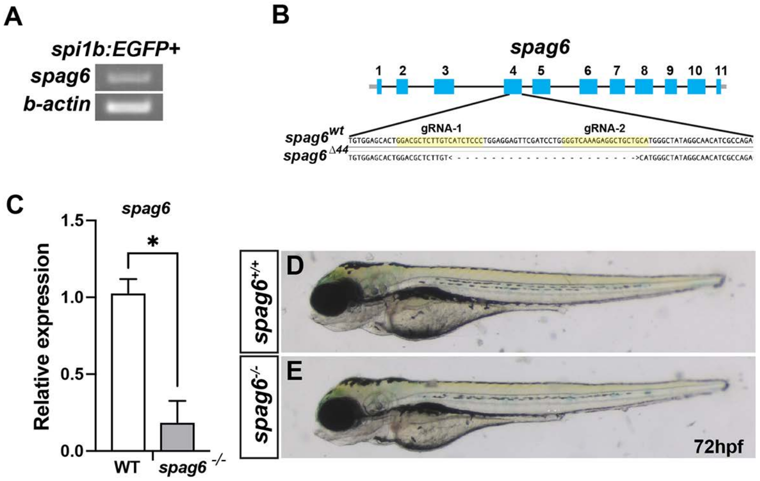

Figure S8. Myeloid expression of *spag6* and CRISPR-Cas9 generated zebrafish *spag6* mutants.

(A) RT-PCR for *spag6* and *β-actin* from *spi1b:GFP*+ cells. (B) Exon schematic and associated gRNA sequences (yellow highlight) used to generate *spag6*$^{-/-}$ mutants. Alignments indicate 44 bp deletion created relative to the WT sequence. (C) RT-qPCR data for *spag6* in WT and *spag6*$^{-/-}$ mutant embryos. * - p<0.05. (D,E) Lateral views of *spag6*$^{+/+}$ and *spag6*$^{-/-}$ embryos at 72hpf.

Disease Models & Mechanisms • Supplementary information

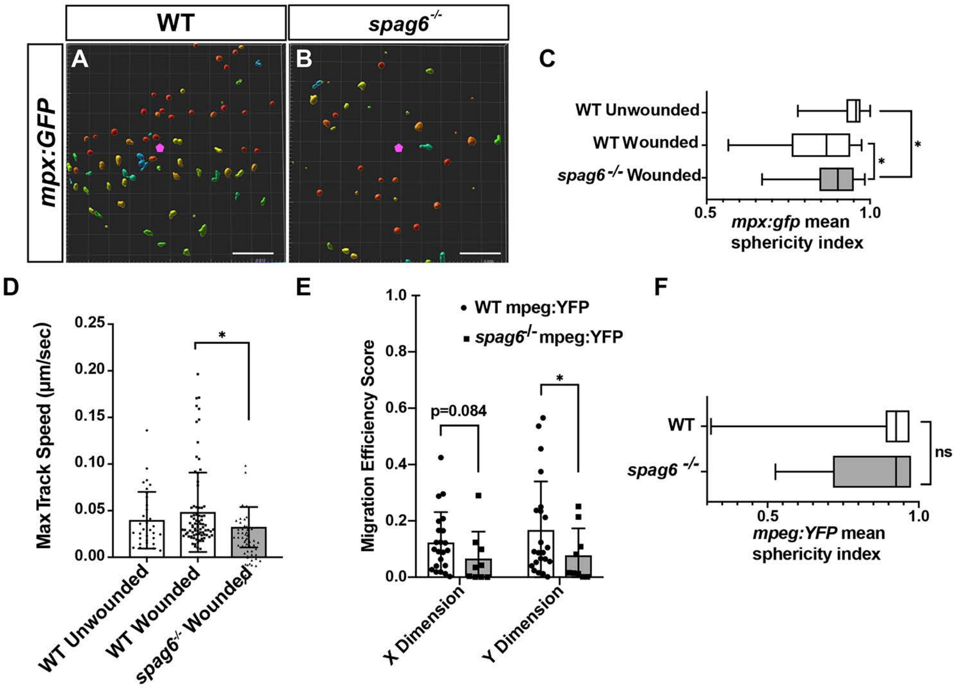

Figure S9. *Spag6* mutant neutrophils and macrophages display altered cell morphology and decreased track speed in response to sterile wounds.

(A,B) Sphericity-coded projections of *mpx:GFP*+ cell surfaces from WT and *spag6*$^{-/-}$ embryos. (C) Quantification of sphericity in WT controls, wounded WT, and *spag6* mutant *mpx:GFP* embryos. (D) Track speed maxima from WT and *spag6*$^{-/-}$ *mpx:GFP* migration tracks. (E) Quantification of migration efficiency scores for WT and *spag6*$^{-/-}$ *mpeg:YFP* embryos calculated from point position data generated in Imaris. Each data point represents an individual cell from a minimum of 3 separate experiments, per genotype. (F) Mean cell sphericity indices for WT and *spag6*$^{-/-}$ *mpeg:YFP* embryos as calculated in Imaris. For C, D, and E, * - $p<0.05$.Scale bars: 100 µm.

Disease Models & Mechanisms • Supplementary information

Table S1. Primers used.

gene	***genotyping primer***	***sequence***
smh	forward	ACGAAACTTGGCATCATTTTCCCACT
smh	reverse	GCAGGAATGGAGAACTCTGA
spag6_t2F	forward	GGGATATGAAAGACAGCGATTC
spag6_t3R	reverse	GGGCAACATTACCGTAAACATT
gene	***RT PCR primer***	***sequence***
ccdc103_dr_F	forward	TGAACCAAAAGGTCGCCAGC
ccdc103_dr_R	reverse	GGTGAACCAGAGATGTCGTTTCG
gata1	forward	AAGATGGGACAGGCCACTAC
gata1	reverse	TGCTGACAATCAGCCTCTTTT
spag6_rt_F	forward	GGGATATGAAAGACAGCGATTC
spag6_rt_R	reverse	GGGCAACATTACCGTAAACATT
bactin	forward	TACAGCTTCACCACCACAGC
bactin	reverse	AGGAAGGAAGGCTGGAAGAG
GAPDH	forward	AATCCCATCACCATCTTCCA
GAPDH	reverse	TGGACTCCACGACGTACTCA
CCDC103_hs_F	forward	GGAGTTCAGGGGTATTGTCCTTG
CCDC103_hs_R	reverse	GTTCCAGGGCACAGTTCTCTTTC
gene	***PPI cloning primer***	***sequence***
ccdc103_hs_attb1_Ct_full_F	forward	GGGGACAAGTTTGTACAAAAAAGCAGGCTTCACCATGGTTGCTAGGCAACCACAGCT
ccdc103_hs_attb2_Ct_full_R	reverse	GGGGACCACTTTGTACAAGAAAGCTGGGTGTAAATTGCCTTGCACTTGGAA
ccdc103_hs_attb1_Nt_full_F	forward	GGGGACAAGTTTGTACAAAAAAGCAGGCTTCGTTGCTAGGCAACCACAGCT
ccdc103_hs_attb2_Nt_full_R	reverse	GGGGACCACTTTGTACAAGAAAGCTGGGTTTTAAAATTGCCTTGCACTTGGAA
spag6-attB2-F1	forward	GGGGACAGCTTTCTTGTACAAAGTGGATTGCAAGACATAATGCAGAACTG
spag6-attB2-R1	reverse	GGGGACCACTTTGTACAAGAAAGCTGGGTtGTTATTAAGTGGTTGATAGCT
dync1h1-attB2-F1	forward	GGGGACAGCTTTCTTGTACAAAGTGGGCCCAGTCCATTTATGGCGGGCGC
dync1h1-attB2-R1	reverse	GGGGACCACTTTGTACAAGAAAGCTGGGTtCTCTGTGCACAAGACTGCGAC
ccdc103_A461C_PX_1_F	forward	GCACTGGCTGATCCCGTGGGGCCGGC
ccdc103_A461C_PX_1_R	reverse	GCCGGCCCCACGGGATCAGCCAGTGC
ccdc103_A461C_PX_2_F	forward	GGTGGCACTGGCTGATCCCGTGGGGCCGGCTGAC
ccdc103_A461C_PX_2_R	reverse	GTCAGCCGGCCCCACGGGATCAGCCAGTGCCACC
ccdc103_G31C_PX_1_F	forward	CATCATCAACTTCAAGCCTTTGGAGAAAGAGC
ccdc103_G31C_PX_1_R	reverse	GCTCTTTCTCCAAAGGCTTGAAGTTGATGATG
ccdc103_G31C_PX_2_F	forward	CATCATCAACTTCAAGCCTTTGGAGAAAGAGCTG
ccdc103_G31C_PX_2_R	reverse	CAGCTCTTTCTCCAAAGGCTTGAAGTTGATGATG
gRNA oligo		***sequence***
spag6-1		GCGTAATACGACTCACTATAGGACGCTCTTGTCATCTCCCGTTTTAGAGCTAGAAATAGC
spag6-2		GCGTAATACGACTCACTATAGGGTCAAAGAGGCTGCTGCAGTTTTAGAGCTAGAAATAGC

Table S2. Antibodies used.

Antibody	***Catalogue #***	***Manufacturer***	***Antibody class***
Ccdc1033-1st cycle-YZ6719	custom - no cat#	Yenzyme	Primary
anti-alpha-Tubulin	T6199	Sigma	Primary
Monoclonal anti -dynein (heavy chain) antibody produced in mouse	D1667-0.2ML	Sigma	Primary
Acetyl-alpha Tubulin (Lys40) Monoclonal (6-11B-1)	32-2700	Thermo-Fisher Scientific	Primary
anti-GFP chicken IgY	A10262	Life Technologies	Primary
Goat anti chicken IgY(H+L) - AlexaFluor 488	6100-30	Southern Biotech	secondary
DAPI (4',6-Diamidino-2-Phenylindole, Dihydrochloride)	D1306	Life Technologies	secondary
Goat anti-mouse IgG2a Alexa Fluor 555	A11120	Life Technologies	secondary
Goat anti-mouse IgG1 AlexaFluor 647	927057	Life Technologies	secondary
Goat anti-mouse IgG2b AlexaFluor 555	A-21147	Life Technologies	secondary

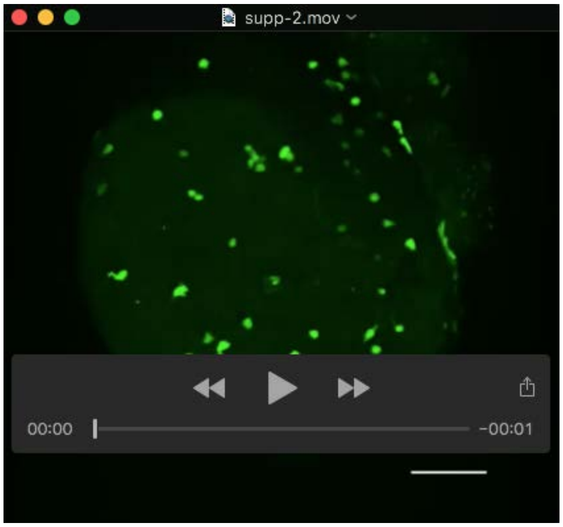

Movie 1. WT - *mpx:GFP* wounding assay.

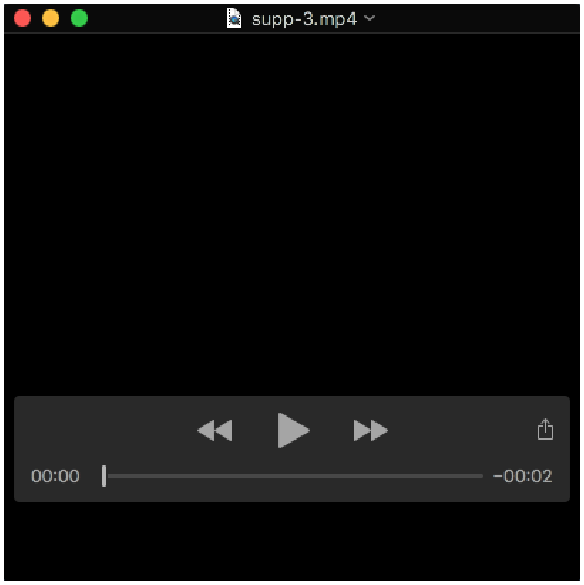

Movie 2. *smh* mutant - *mpx:GFP* wounding assay.

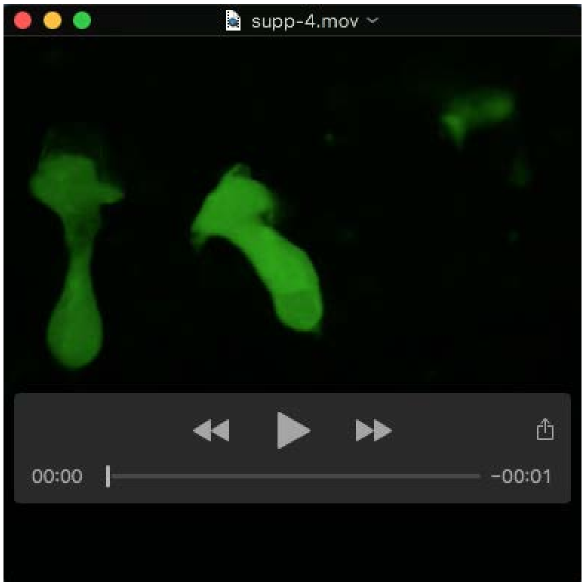

Movie 3. WT - *mpx:GFP+* cell, 60X Objective.

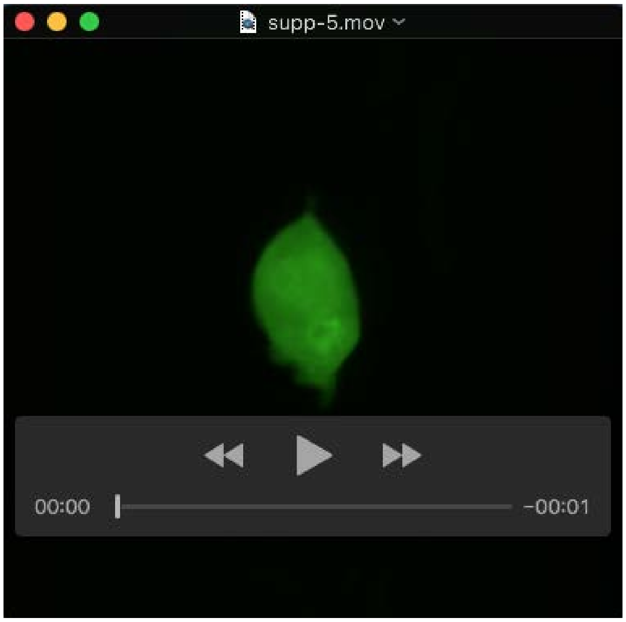

Movie 4. *smh* mutant - *mpx:GFP+* cell, 60X Objective.

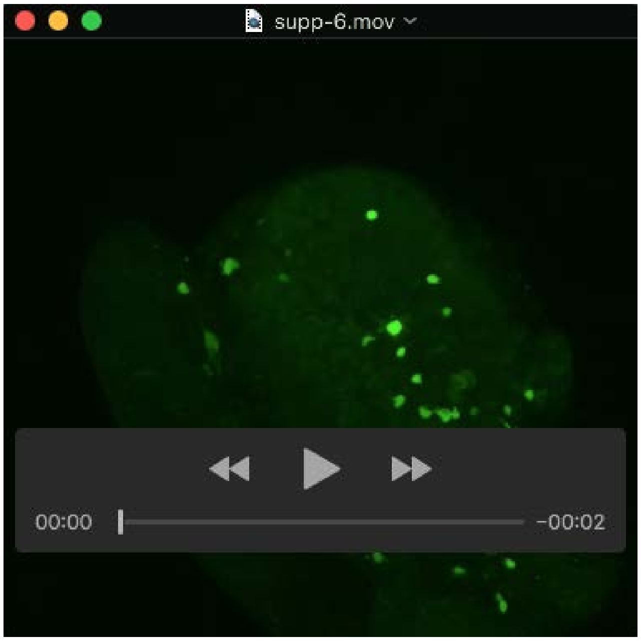

Movie 5. WT - *mpeg:GFP* wounding assay.

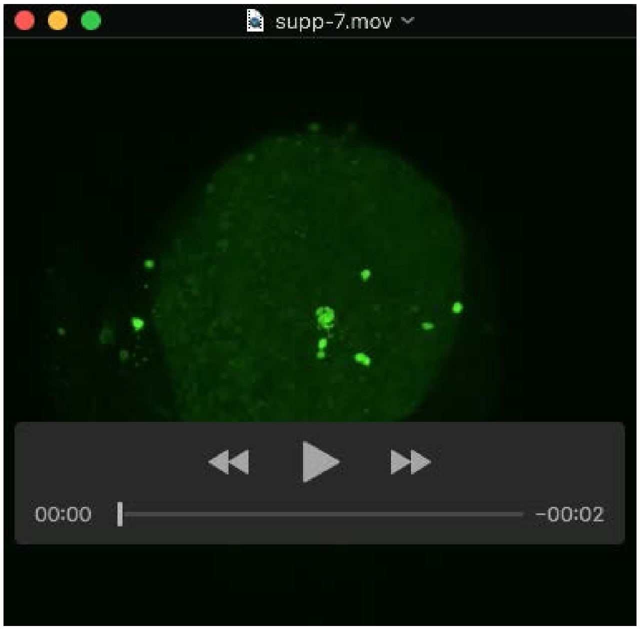

Movie 6. *smh* mutant - *mpeg:GFP* wounding assay.

Disease Models & Mechanisms • Supplementary information

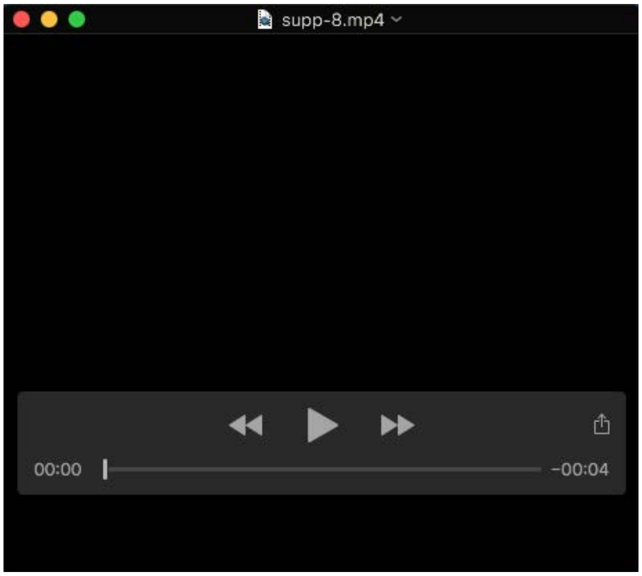

Movie 7. WT - *mpx:GFP* + paclitaxel in wounding assay.

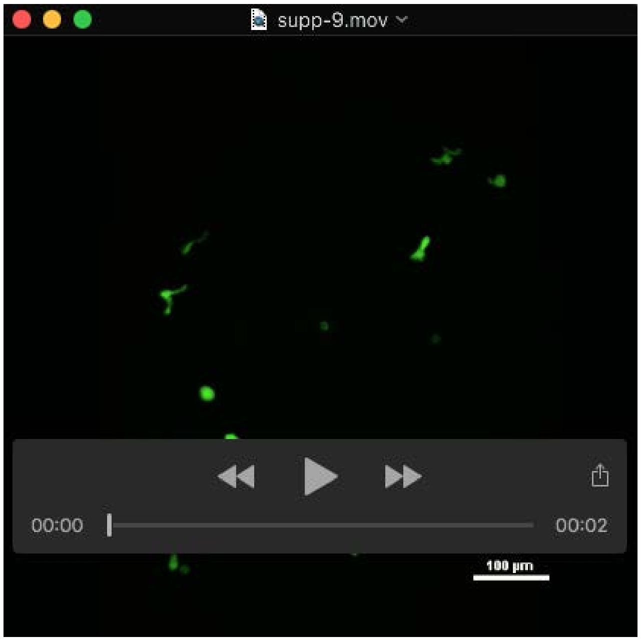

Movie 8. *smh* - *mpx:GFP* + paclitaxel in wounding assay.

Disease Models & Mechanisms • Supplementary information

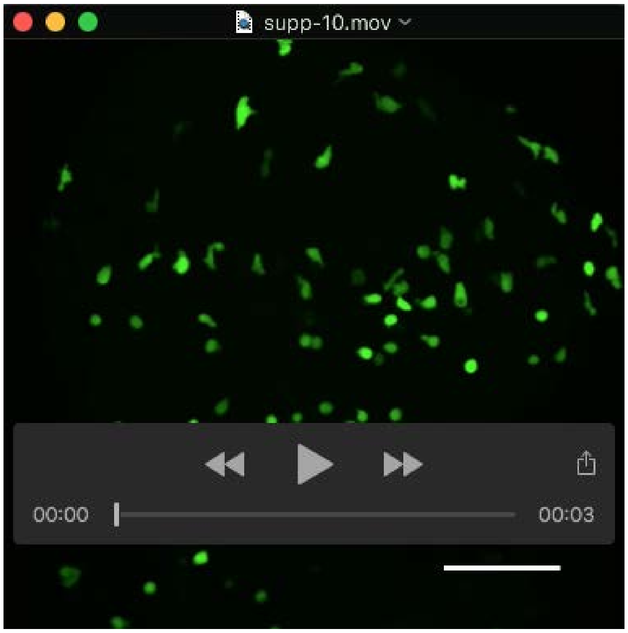

Movie 9. WT (*spag6*)- *mpx:GFP* wounding assay.

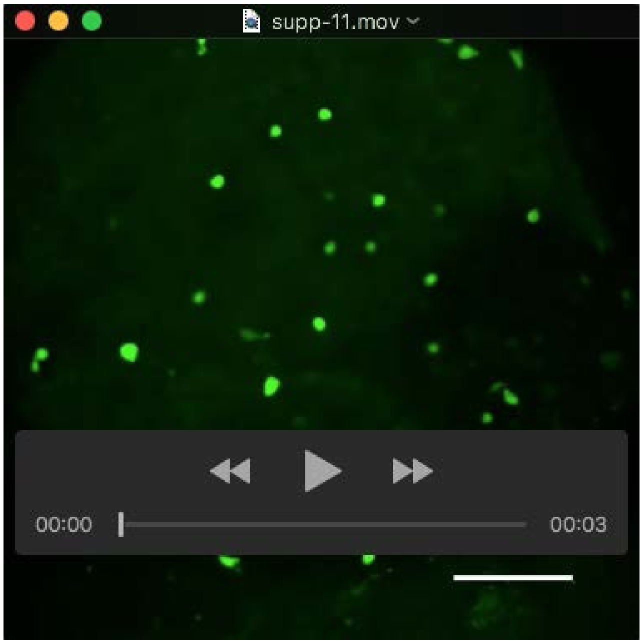

Movie 10. *spag6* mutant - *mpx:GFP* wounding assay.

Disease Models & Mechanisms • Supplementary information
